# Comparison of Two *Aspergillus oryzae* Genomes From Different Clades Reveals Independent Evolution of Alpha-Amylase Duplication, Variation in Secondary Metabolism Genes, and Differences in Primary Metabolism

**DOI:** 10.3389/fmicb.2021.691296

**Published:** 2021-07-13

**Authors:** Katherine Chacón-Vargas, Colin O. McCarthy, Dasol Choi, Long Wang, Jae-Hyuk Yu, John G. Gibbons

**Affiliations:** ^1^Molecular and Cellular Biology Graduate Program, University of Massachusetts, Amherst, MA, United States; ^2^Department of Food Science, University of Massachusetts, Amherst, MA, United States; ^3^Deapertment of Food Science, University of Wisconsin-Madison, Madison, WI, United States; ^4^Department of Bacteriology, and Food Research Institute, University of Wisconsin-Madison, Madison, WI, United States; ^5^State Key Laboratory of Mycology, Institute of Microbiology, Chinese Academy of Sciences, Beijing, China; ^6^Department of Systems Biotechnology, Konkuk University, Seoul, South Korea; ^7^Organismic & Evolutionary Biology Graduate Program, University of Massachusetts, Amherst, MA, United States

**Keywords:** *Aspergillus oryzae*, comparative genomics, Oxford Nanopore sequencing, fermentation, amylase, Koji

## Abstract

Microbes (bacteria, yeasts, molds), in addition to plants and animals, were domesticated for their roles in food preservation, nutrition and flavor. *Aspergillus oryzae* is a domesticated filamentous fungal species traditionally used during fermentation of Asian foods and beverage, such as sake, soy sauce, and miso. To date, little is known about the extent of genome and phenotypic variation of *A. oryzae* isolates from different clades. Here, we used long-read Oxford Nanopore and short-read Illumina sequencing to produce a highly accurate and contiguous genome assemble of *A. oryzae* 14160, an industrial strain from China. To understand the relationship of this isolate, we performed phylogenetic analysis with 90 *A. oryzae* isolates and 1 isolate of the *A. oryzae* progenitor, *Aspergillus flavus*. This analysis showed that *A. oryzae* 14160 is a member of clade A, in comparison to the RIB 40 type strain, which is a member of clade F. To explore genome variation between isolates from distinct *A. oryzae* clades, we compared the *A. oryzae* 14160 genome with the complete RIB 40 genome. Our results provide evidence of independent evolution of the alpha-amylase gene duplication, which is one of the major adaptive mutations resulting from domestication. Synteny analysis revealed that both genomes have three copies of the alpha-amylase gene, but only one copy on chromosome 2 was conserved. While the RIB 40 genome had additional copies of the alpha-amylase gene on chromosomes III, and V, 14160 had a second copy on chromosome II and an third copy on chromosome VI. Additionally, we identified hundreds of lineage specific genes, and putative high impact mutations in genes involved in secondary metabolism, including several of the core biosynthetic genes. Finally, to examine the functional effects of genome variation between strains, we measured amylase activity, proteolytic activity, and growth rate on several different substrates. RIB 40 produced significantly higher levels of amylase compared to 14160 when grown on rice and starch. Accordingly, RIB 40 grew faster on rice, while 14160 grew faster on soy. Taken together, our analyses reveal substantial genome and phenotypic variation within *A. oryzae*.

## Introduction

Domestication is an evolutionary process that involves the genetic modification of a species by breeding it in isolation from its ancestral population in an effort to enhance its utility to humans (Larson et al., 2014). Early farmers used selective breeding to continuously cross individuals with desired traits, eventually yielding crops with more food (i.e., larger/more seeds and fruits) that were easier to harvest (i.e., loss of seed shattering in grains), and livestock that were less aggressive and more fertile (Purugganan and Fuller, 2009; Abbo et al., 2014; Larson and Fuller, 2014; Larson et al., 2014). Domestication can also lead to sub specialization and genetic divergence of lineages within a given domesticated species as observed in chickens, which were independently bred for meat and for eggs (Rubin et al., 2010).

In parallel with plants and animals, microbes (bacteria, yeasts, and molds) were also domesticated, most likely because of their role in food preservation (Gibbons and Rinker, 2015; Steensels et al., 2019). Archeological chemistry evidence of ruminant dairy fat from ∼8,000 year old pottery shards in Northern Europe suggest that humans were employing microbial fermentation to produce cheese (Salque et al., 2013). Similarly, the chemical signatures of a “proto rice wine” were discovered embedded in ∼9,000 year old pottery shards from China (McGovern et al., 2004). Further, the long-term relationship between humans and fungi used for food fermentation is evident through the analysis of archeological remains using (i) ancient DNA sequencing (Cavalieri et al., 2003), (ii) microscopy paired with morphological characterization (Liu et al., 2019) and, (iii) direct isolation of characterization of microbial specimens (Aouizerat et al., 2019, 2020).

*Saccharomyces* yeasts are the most thoroughly studied domesticated fungi. In particular, there are a number of domesticated lineages of *Saccharomyces cerevisiae* that have been shaped by artificial selection for particular fermentation applications. For example, lineages of beer yeasts have an increased capacity to metabolize maltotriose (a highly abundant sugar in wort) while also producing fewer off-flavor compounds like 4-vinyl guaiacol (Gallone et al., 2016). In addition to yeasts, several filamentous fungi have been domesticated. For instance, the white mold *Penicillium camemberti* was domesticated for its role in the maturation of soft cheeses (Ropars et al., 2020). Artificial selection in *P. camemberti* resulted in white color, increased aerial growth, reduced toxin production, and increased inhibition of fungal competitors compared to its progenitor (Ropars et al., 2020). Additionally, *Penicillium roqueforti* was domesticated for the production of blue cheeses like Roquefort (Dumas et al., 2020). Two distinct lineages of *P. roqueforti* exist that are associated with pre-industrial and industrial cheese production, and possess beneficial traits for these usages.

*Aspergillus oryzae* is a domesticated filamentous fungal species used in the production of traditionally fermented Asian foods and beverages, such as shoyu, miso, sake, and meju (Machida et al., 2005, 2008; Gibbons et al., 2012; Alshannaq et al., 2018; Watarai et al., 2019). *A. oryzae* was domesticated from *Aspergillus flavus* (Geiser et al., 1998; Machida et al., 2005; Gibbons et al., 2012), or perhaps the closely related species *Aspergillus aflatoxiformans* or *Aspergillus minisclerotigenes* (Kjaerbolling et al., 2020). As a result of domestication and specialization to the fermented food environment, *A. oryzae* has reduced capacity to produce many secondary metabolites like aflatoxin and cyclopiazonic acid, and increased carbohydrate metabolism, in part due to the duplication of the alpha-amylase encoding gene (Machida et al., 2005; Hunter et al., 2011; Gibbons et al., 2012; Nemoto et al., 2012). Recently, Watarai et al. (2019) sequenced and analyzed the genomes of 82 *A. oryzae* strains, and identified eight distinct clades. However, little is known about the genome and functional divergence between these *A. oryzae* groups.

In this study, we used a combination of short-read and long-read DNA sequencing to assemble a highly contiguous genome of the clade A isolate *A. oryzae* 14160, originally isolated from China. To gain insight into *A. oryzae* genome variation, we compared the *A. oryzae* 14160 genome to the *A. oryzae* RIB 40 (clade F) reference genome. We also examined phenotypic differences between the two isolates by measuring amylase activity and growth rate on several culture medias. Our results show that *A. oryzae* 14160 and RIB 40 differ substantially in terms of their genomes and phenotypes.

## Materials and Methods

### Isolates, Fungal Culturing, and DNA Extraction

*A. oryzae* 14160 was originally isolated from Xinyang City, Henan Province, China. Spores were cultured in potato dextrose agar (PDA) at 30°C for 48 h. DNA was extracted directly from spores following the protocol from Lee et al. (2017). Qubit and Nanodrop were used to quantify DNA.

### Illumina and Oxford Nanopore Sequencing

PCR-free Illumina libraries were constructed and sequenced by Novogene. Illumina sequencing was conducted in paired-end 150 bp format. Raw reads were deduplicated by Tally using “with-quality” and “pair-by-offset” parameters to remove exact paired-end duplicates (Davis et al., 2013). Deduplicated reads were then trimmed with Trim Galore^1^ using “stringency 1,” “quality 30,” and “length 50” parameters to remove any adaptor sequences and low quality positions. Error correction was performed using the default settings in SPAdes (Bankevich et al., 2012). Data quality was assessed using FASTQC^2^.

Oxford Nanopore (ONT) libraries were prepared using 400 ng of gDNA following the manufacturer’s instructions with the 1D Rapid Sequencing Kit (SQK-NSK007). ONT sequencing was performed on a MinION following the manufacturer’s instructions. The *A. oryzae* 14160 library was run for 12 h. ONT reads were mapped against the *A. oryzae* 14160 genome assembly to assess quality of reads using minimap2 (Li, 2018). Raw Illumina and ONT data for *A. oryzae* 14160 are available through the NCBI SRA under BioProject accession number PRJNA717291.

### Phylogenetic Analysis of *A. oryzae* 14160

To reconstruct the evolutionary history of *A. oryzae* 14160 we analyzed the phylogenetic relationship of 89 *A. oryzae* strains from Gibbons et al. (2012) and Watarai et al. (2019) as well as the *A. oryzae* RIB 40 and *A. flavus* 3357 reference genomes (Machida et al., 2005; Nierman et al., 2015). BioProject accession numbers for samples are as follows: PRJDB7763 for TK-10, TK-11, TK-12, TK-13, TK-14, TK-15, TK-16, TK-17, TK-18, TK-19, TK-1, TK-20, TK-21, TK-22, TK-23, TK-24, TK-25, TK-26, TK-27, TK-28, TK-29, TK-2, TK-30, TK-31, TK-32, TK-33, TK-34, TK-35, TK-36, TK-37, TK-38, TK-39, TK-3, TK-40, TK-41, TK-42, TK-43, TK-44, TK-45, TK-46, TK-47, TK-48, TK-49, TK-4, TK-50, TK-51, TK-52, TK-53, TK-54, TK-55, TK-56, TK-57, TK-58, TK-59, TK-5, TK-60, TK-61, TK-62, TK-63, TK-64, TK-65, TK-66, TK-67, TK-68, TK-69, TK-6, TK-70, TK-71, TK-72, TK-73, TK-74, TK-75, TK-76, TK-77, TK-78, TK-79, TK-7, TK-80, TK-81, TK-82, TK-8, and TK-9, and PRJNA164603 for AO_302 (SRRC 302), AO_331 (RIB 331), AO_333 (RIB 333), AO_537 (RIB 537), AO_632 (RIB 632), AO_642 (RIB 642), and AO_949 (RIB 949). First, Illumina whole-genome data was de-duplicated, and adapter and quality trimmed as described above. Next, sequence reads from each isolate were mapped to the *A. oryzae* RIB40 reference genome with BWA-MEM v0.7.15 (Li and Durbin, 2009, 2010). SAM files were converted into sorted BAM format using the samtools v1.4.1 “view” and “sort” option (Li et al., 2009). Variant calling was performed with GATK using the “Germline short variant discovery” best practices pipeline (McKenna et al., 2010). The GATK “Haplotype Caller” option was used to call SNPs. Genotype calls for the 92 samples were combined using the “GenotypeVCFs” option. SNPs were extracted and filtered using the “SelectVariants” and “VariantFiltration” options. SNP filtering was performed using “hard filtering” with the parameters: “QD < 21.0 | | FS > 0.5 | | MQ < 60.0 | | MQRankSum < −0.2 | | ReadPosRankSum < −4.0 | | SOR > 1.0.” Phylogenetic analysis was performed with RAxML (Stamatakis, 2014) with the GTRGAMMA model and 100 bootstrap replicates. The phylogenetic tree was visualized with ggtree (Yu, 2020) and ggplot2 (Wickham, 2009). Phylogenetic analysis was conducted independently with alignments of 243,486, 7,641, and 3,340 SNPs. Phylogenetic trees generated from reduced SNP marker alignments were performed to investigate the impact of linkage on the inferred phylogenetic relationship. SNPs were separated by a minimum of 4 and 10 Kb for the 7,641, and 3,340 SNP marker sets, respectively.

### Genome Assembly and Annotation

The *A. oryzae* 14160 genome was assembled using a hybrid approach that combined the short-read Illumina and long-read ONT data. Error correction and genome assembly was performed with the MaSuRCa assembler with the default parameters (Zimin et al., 2013). The quality of the *A. oryzae* 14160 genome assembly was assessed with QUAST (Gurevich et al., 2013) and genome completeness was evaluated with BUSCO (Simao et al., 2015).

Gene prediction and annotation of *A. oryzae* 14160 strain were performed using the Funannotate pipeline^3^. Gene model prediction was evaluated with BUSCO (Simao et al., 2015). Functional annotation was performed with Interproscan 5 (Jones et al., 2014) using the default settings and complemented with Phobius (Kall et al., 2004, 2007) for transmembrane topology and signal peptide prediction. Finally, secondary metabolism associated gene clusters were predicted using antiSMASH using the “strict” detection strictness setting (Medema et al., 2011). The *A. oryzae* 14160 genome assembly is available through the BioProject accession number PRJNA717291.

### Whole Genome Alignment

MUMer was used to align the *A. oryzae* 14160 assembly to the RIB 40 reference genome using the parameters “-mum,” “-b,” and “-c” (Delcher et al., 1999). The Nucmer alignment tool was used to identify conserved synteny between the *A. oryzae* 14160 and RIB 40 genomes using the “-maxmatch” and “-c 1000” parameters. Nucmer output was filtered using the Delta-Filter tool from with the parameter “-I 4000.” Alignment coordinates were extracted by the “show-coords” function from MUMmer using the “-r”, “-c,” and “−l” parameters. Whole genome synteny was visualized using Circos (Krzywinski et al., 2009).

### Alpha-Amylase Locus Synteny Analysis

*A. oryzae* isolates possess between 1 and 4 copies of the alpha-amylase encoding gene (Watarai et al., 2019). We compared the alpha-amylase encoding genes and their flanking regions between *A. oryzae* RIB 40 and *A. oryzae* 14160 to determine whether the duplication events shared an evolutionary history, or evolved independently. To identify the alpha-amylase encoding genes in *A. oryzae* 14160 we used BLASTN with the *A. oryzae* RIB 40 *amy1* gene (*AO090023000944*) as the query and *A. oryzae* 14160 predicted transcripts as the subject, with an *e*-value cutoff of 1e-30 (Altschul et al., 1997). We also conducted this BLASTN search with the *A. oryzae* 14160 genome assembly. These searches yielded three independent loci containing the alpha-amylase encoding gene in the *A. oryzae* 14160 genome. Finally, the Funannotate annotation was used for validation (each of the three alpha-amylase copies in the *A. oryzae* 14160 genome were annotated as “AMY1”).

Next, we used SimpleSynteny to visualize the synteny between *A. oryzae* RIB40 and the *A. oryzae* 14160 alpha-amylase loci, using the default settings (Veltri et al., 2016). To increase confidence that our results were not the byproduct of assembly errors, we also assembled the *A. oryzae* 14160 genome with Canu (ONT data only) and SPAdes (ONT and Illumina data), using the default settings (Bankevich et al., 2012; Koren et al., 2017). We reasoned that independent misassembles of identical loci would be exceedingly rare. Synteny analysis of each of the three *A. oryzae* 14160 alpha-amylase loci (including five genes upstream and five genes downstream of the alpha-amylase encoding genes) were visualized between the MaSuRCA (primary assembly), Canu, and SPAdes assemblies and the *A. oryzae* RIB 40 reference genome. Additionally, because some alpha-amylase loci in *A. oryzae* contain the a transposable element likely responsible for duplications, we used BLASTN to search for the presence of the Tc1/Mariner class putative transposable element (NCBI accession AB072434.1) in the *A. oryzae* 14160 genome using an *e*-value cutoff of 1e-30. Finally, to provide further evidence for accurate assembly of the alpha-amylase loci, we used BLASTN searches to identify long ONT reads that spanned the alpha-amylase gene and flanking genes that differentiated each *A. oryzae* 14160 alpha-amylase loci using an *e*-value cutoff of 1e-30 and a query coverage cutoff of 80%.

### Alpha-Amylase Upstream Sequence Analysis

We aligned the 1 Kb upstream region of each of the six alpha-amylase genes to explore whether divergence between gene copies or strains correlated with amylase activity. Bedtools was used to extract the 1 Kb upstream region from each alpha-amylase locus (Quinlan and Hall, 2010). The alignment was performed with MAFFT with the following parameters: “–kimura 1,” “–op 3.0,” and “–ep 0.5” (Katoh and Standley, 2013).

### Single Nucleotide Polymorphism Analysis

We predicted single nucleotide polymorphisms (SNPs) in *A. oryzae* 14160 vs. the *A. oryzae* RIB 40 reference genome (Machida et al., 2005). *A. oryzae* 14160 quality and adapter trimmed and error corrected Illumina reads were mapped against the *A. oryzae* RIB 40 reference genome using BWA-MEM v0.7.15 (Li and Durbin, 2009, 2010). SNPs were called using freebayes v1.3.1 with the default settings with the exception of setting ploidy to haploid (–ploidy = 1) (Garrison and Marth, 2012). Next, we used vcftools v0.1.14 to filter variants with the following parameters “–remove-indels,” “–remove-filtered-all,” “–min-meanDP 25,” “–minQ 20,” “—recode,” and “–recode-INFO-all” (Danecek et al., 2011). SNPs from this filtered VCF file were annotated with SnpEff v4.3t using “Aspergillus_oryzae” as the genome database (Cingolani et al., 2012). Using the SnpEff output, we calculated missense variant rate for each gene to identify genes with relatively elevated occurrences of missense variants. Per gene missense variant rate was calculated as:

(1) $$M\,i\,s\,s\,e\,n\,s\,e\,V\,a\,r\,i\,a\,n\,t\,R\,a\,t\,e=\frac{n\,u\,m\,b\,e\,r\,o\,f\,m\,i\,s\,s\,e\,n\,s\,e\,v\,a\,r\,i\,a\,n\,t\,s}{l\,e\,n\,g\,t\,h\,o\,f\,a\,l\,l\,e\,x\,o\,n\,s}$$

Gene Ontology Enrichment of gene sets with SNP profiles of interest was conducted through the FungiFun2 server^4^, using the default settings (Priebe et al., 2015).

### Identification of Lineage Specific Genes

To identify gene absences specific to the *A. oryzae* 14160 and RIB40 genomes, we used control-FREEC to estimate the copy number of each 1 kb window with a 200 bp step size (Boeva et al., 2012). The following parameters were used: window = 10, telocentromeric = 0, minExpectedGC = 0.33, and maxExpectedGC = 0.63. To estimate CNV for each gene, we used a custom perl script that takes gene coordinates and the control-FREEC output as input (CNV_gene-overlap.pl script is available here: https://github.com/DaRinker/PolarBearCNV) (Rinker et al., 2019). Entire genes (a minimum of start codon to stop codon) with copy numbers of zero when mapped against the non-self genome assembly (i.e., *A. oryzae* RIB 40 vs. *A. oryzae* 14160 reference, or *A. oryzae* 14160 vs. *A. oryzae* RIB 40 reference) were considered lineage specific genes in the reference genome.

### Amylase Activity Assays

We used a quantitative method to measure amylase activity *via* the Megazyme (Bray, Ireland) alpha-amylase Assay Kit (Ceralpha Method). Short grain sushi rice was sterilized and cooked in distilled water at a ratio of 1:1.7 at 121°C for 15 min. Fifteen grams of cooked rice was inoculated with ∼100,000 conidia suspended in 20 μL H_2_O and incubated for 48 h at 32°C. The entire sample was transferred into a 50 ml centrifuge tube, washed with 10 ml distilled H_2_O and vortexed for 1 min. A 2 ml aliquot of wash water was transferred to a 5 ml tube and centrifuged at 1,000 RPM for 10 min. A one mL aliquot of the supernatant was diluted with 49 mL alpha-amylase buffer. Buffered enzyme extract was preheated at 40°C for 5 min after which a 0.1 mL aliquot was added to an equal amount of Megazyme Ceralpha Amylase HR Reagent in triplicate and maintained at 40°C for 10 min. Next, 1.5 mL 1% sodium triphosphate solution was added to halt the reaction. Control samples were prepared by immediately adding the sodium triphosphate solution to the enzyme-substrate solution. The samples and controls were transferred into a 24 well microplate and absorbance was measured at 405 nm.

We also used an iodine-based qualitative assay to examine amylase activity (Fuwa, 1954) while isolates grew on starch agar (per 1 L: 3 g beef extract, 10 g soluble starch and 12 g agar at pH 7.5). In this assay, iodine forms a black/dark blue complex with starch, but does not stain sugars, resulting in a yellowish zone surrounding the colony where starches have been metabolized. ∼10,000 conidia suspended in 20 μL H_2_O were pipetted onto the center of the starch plates. Plates were incubated at 32°C for 39 h then flooded with iodine. This experiment was performed in triplicate. Plates were imaged with the Interscience Scan1200.

### Proteolytic Assay

To examine the proteolytic activity of isolates, we performed an established zone of clearance assay (Rajamani and Hilda, 1987). Briefly, ∼100,000 conidia suspended in 20 μL H_2_O were inoculated onto media consisting of 2.5 g agar, 2.5 g powdered skim milk and 125 mL distilled H_2_O. Ten biological replicates of each strain were grown at 32°C for 72 h, at which time the zone of clearance was measured at two independent locations using digital calipers. The size of the zone is positively associated with higher proteolytic activity (Rajamani and Hilda, 1987). A 2-talied *t*-test was conducted to compare zone of clearance size between isolates.

### Growth Rate of *A. oryzae* 14160 and *A. oryzae* RIB 40

We compared the growth rate of *A. oryzae* 14160 and RIB 40 on starch agar (as defined above), PDA, rice agar and soy agar. PDA (Fisher Scientific DF0013) was prepared according to manufacturer instructions. Rice agar was prepared using 75 g of short grain sushi rice which was powered in a dry blender, and 15 g agar in 1 L distilled H_2_O. For the soybean agar, dried soybeans were soaked in H_2_O for 24 h, then 30 g soybean and 15 g agar were pureed together in 1 L distilled H_2_O. All media was sterilized and cooked *via* autoclaving, and 30 mL of media was plated into petri dishes. 20 μL of 500,000 conidia/mL spore solutions were pipetted onto the center of each plate. All growth rate experiments were performed in 10 replicates. Plates were incubated at 32°C for 39 h. Because colony morphology is not always uniformly circular, colony diameter was measured at two independent points for each colony using digital calipers. The average colony diameter for each plate value was used for the statistical analysis. One-way ANOVA was used to test the null hypothesis that growth rate did not differ between culture media for each isolate. *T*-tests were performed on each culture media to test the null hypothesis that *A. oryzae* 14160 and RIB 40 growth rates did not differ.

## Results

### DNA Sequencing Data

We generate 830,485 ONT reads totaling ∼5 billion bp with an average and median read length of 6,065 and 4,092 bp, respectively, and an N50 value of 10,289 bp (Supplementary Figure 1). 96.69% of ONT reads mapped to the *A. oryzae* 14160 assembly. For Illumina data, a total of 17,286,313 paired-end reads were retained after adapter trimming, quality trimming, and error correction.

### Phylogenetic Analysis

**FIGURE 1:**
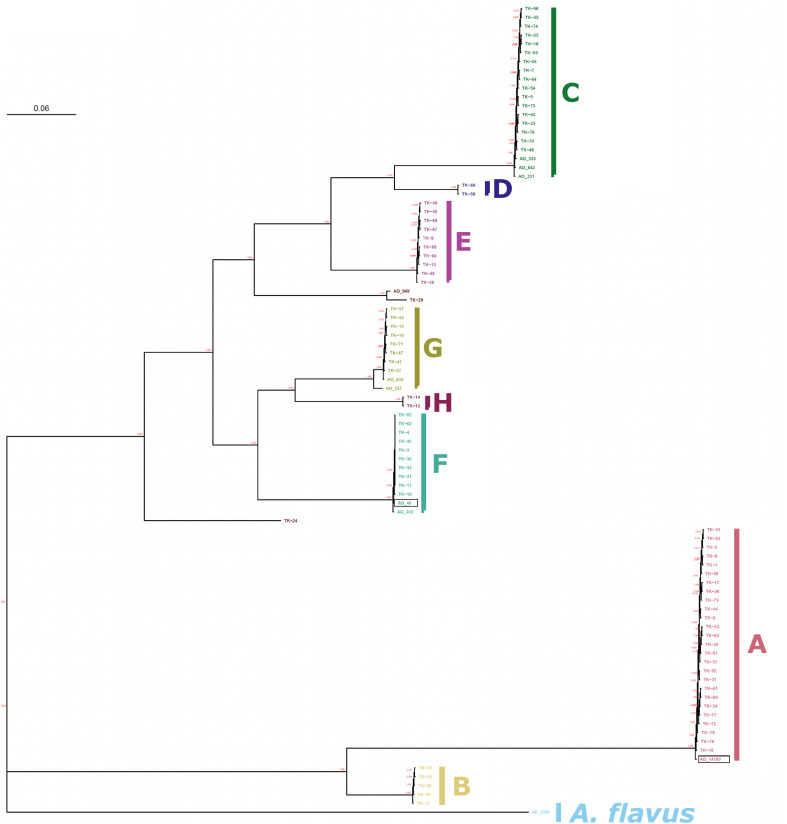
Phylogenetic relationship of *A. oryzae* 14160 and RIB 40. Maximum-likelihood phylogeny of 91 *A. oryzae* isolates and *A. flavus* NRRL 3357. Clades are labeled with respect to the nomenclature used by Watarai et al. (2019). *A. oryzae* 14160 and *A. oryzae* RIB 40 are outlined with a black back and belong to clades F and A, respectively.

We performed phylogenetic analysis to investigate the relationship of *A. oryzae* 14160 in relation to the eight major clades of *A. oryzae* (Watarai et al., 2019). Specifically, we identified 243,486 SNPs from publicly available Illumina whole-genome sequencing data from 91 *A. oryzae* isolates and *A. flavus* NRRL 3357 (Gibbons et al., 2012; Nierman et al., 2015; Watarai et al., 2019) (see Methods for NCBI BioProject accession numbers). A phylogenetic tree was inferred from the alignment of SNPs with RAxML (Stamatakis, 2014) and the tree was rooted by *A. flavus* NRRL 3357. Our results were identical with Watarai et al. (2019) in showing that *A. oryzae* isolates group into eight major clades (A-H) (Figure 1). *A. oryzae* 14160 was nested within clade A, which contained 26 other *A. oryzae* isolates from Japan (Watarai et al., 2019), and *A. oryzae* RIB 40 was nested within clade F (Figure 1). To explore the impact of SNP marker linkage on inferred evolutionary relationship of isolates, we also conducted phylogenetic analysis using smaller subsets of SNP markers separated by a minimum physical distance of 4 and 10 Kb. For all analyses, the clade compositions were identical (Figure 1 and Supplementary Figures 2, 3).

### *A. oryzae* 14160 Genome Assembly and Annotation

*De novo* hybrid genome assembly of *A. oryzae* 14160 was performed with the MaSuRCA assembler (Zimin et al., 2013). The *A. oryzae* 14160 genome was assembled into 24 scaffolds with a cumulative length of 36.5 Mb, largest scaffold length of 4.15 Mb, an N50 of 2.21 Mb and an N90 of 937 Kb. Genome assessment was performed with Quast (Gurevich et al., 2013) and showed a 95% genome fraction compare to the reference *A. oryzae* RIB40 genome. Genome completeness was evaluated with BUSCO (Simao et al., 2015) and showed 99% recovery of complete BUSCO genes with (0.1% fragmented BUSCO genes and 0.9% missing BUSCO genes). Both analyses indicate that the *A. oryzae* 14160 genome assembly is of high quality in terms of contiguity and accuracy.

Genome prediction and annotation of *A. oryzae* 14160 was performed using the Funannotate pipeline which relies on Augustus for gene prediction (Stanke and Waack, 2003). Using this pipeline, we predicted 11,852 protein-coding genes in *A. oryzae* 14160, which is similar to the RIB 40 genome (12,074 protein-coding genes). The gene set was assessed for completeness using BUSCO, resulting in 93% completeness with only 5% fragmented genes and 1.7% missing genes.

### *A. oryzae* 14160 and RIB40 Chromosomal Alignment

**FIGURE 2:**
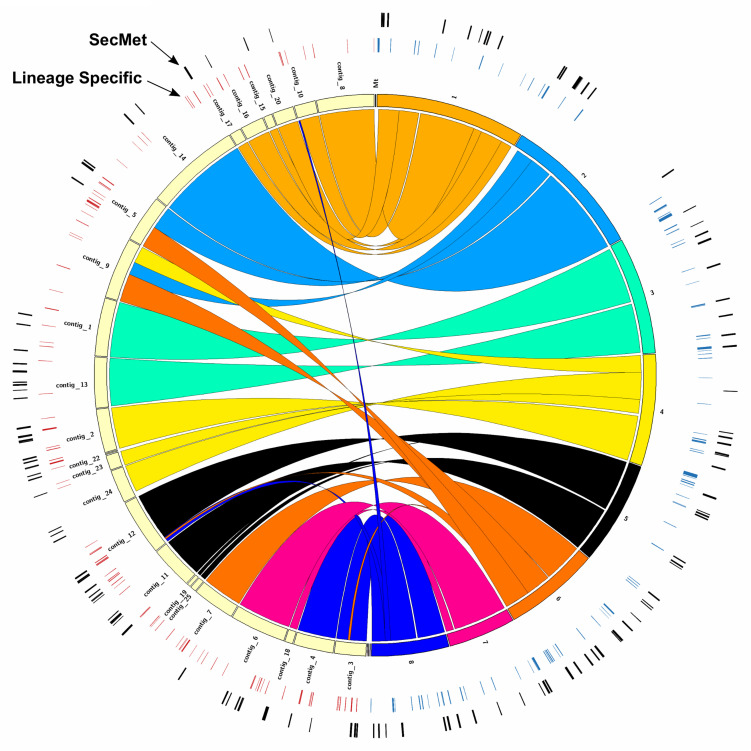
Genome architecture between *A. oryzae* 14160 and RIB 40. Circos plot displaying similarity between the *A. oryzae* 14160 (left half of circle) and RIB 40 (right half of circle) genomes. The innermost ring represents *A. oryzae* 14160 scaffold ID or *A. oryzae* RIB 40 chromosome ID, and colored regions connecting the two represent regions with high sequence similarity (≥95%, length ≥ 10 Kb). The outer circles represent lineage specific genes and genes located within putative secondary metabolite encoding gene clusters (SecMet). The three small contigs to the left of *A. oryzae* RIB 40 chromosome 8 are AP007160, AP007177, and AP007156 (from left to right).

We used Mummer to investigate the synteny between the 24 *A. oryzae* 14160 scaffolds and the eight *A. oryzae* RIB 40 chromosomes. contig_8, contig_10, contig_20, contig_15, contig_16, and contig_7 mapped to chromosome 1, contig_14 and part of contig_5 mapped to chromosome 2, contig_1 and contig_13 mapped to chromosome 3, contig_2, contig_22, contig_23, and contig_24 mapped to chromosome 4, contig_12, contig_11, contig_19, and contig_25 mapped to chromosome 5, contig_7 mapped to chromosome 6, contig_6 and contig_18 mapped to chromosome 7, and contig_4 and contig_3 mapped to chromosome 8 (Figure 2). Nearly all contigs from *A. oryzae* 14160 mapped uniquely to their respective RIB40 chromosome with exception of contig_5 and contig_9, which aligned to multiple chromosomes (Figure 2).

### Alpha-Amylase Synteny

**FIGURE 3:**
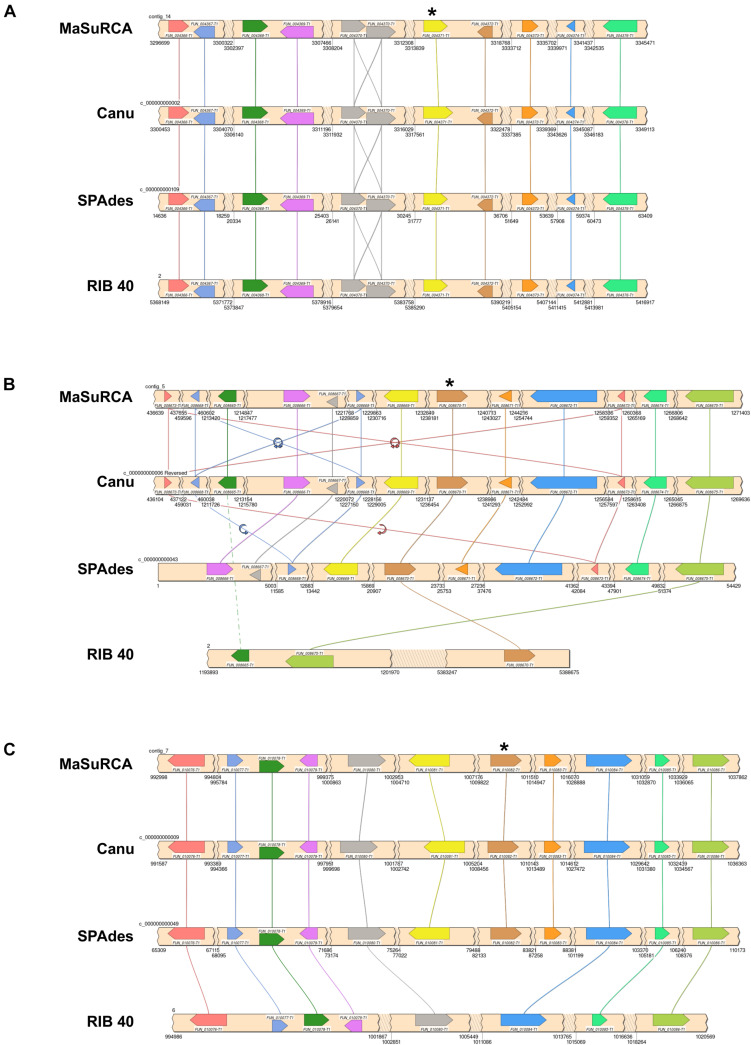
Independent evolution of alpha-amylase duplication in *A. oryzae* 14160. Synteny analysis of the conserved *A. oryzae* 14160 alpha-amylase locus on chromosome 2 **(A)**, the non-syntenic alpha-amylase locus on chromosome 2 **(B)**, and the non-syntenic alpha-amylase locus on chromosome 6 **(C)** from three independent genome assemblies of *A. oryzae* 14160 (MaSuRCA, Canu, and SPAdes) and the reference RIB 40 genome. For each locus, gene direction is indicated by pointed ends, jagged edges indicate a genomic region without protein-coding genes that is skipped for figure clarity. Gene names are with respect to the primary *A. oryzae* 14160 annotation [generated from the MaSuRCA assembly (Zimin et al., 2013) and annotated with the Funannotate pipeline]. Scaffold ID or chromosome number and coordinates are labeled. “*” represents the alpha-amylase encoding genes. Lines connecting genes indicated conservation, and flip arrows represent a change in gene direction **(B)**. Locus schematics were generated with SimpleSynteny (Veltri et al., 2016).

We identified three distinct loci containing the alpha-amylase encoding gene in the *A. oryzae* 14160 genome (Figure 3) (alpha-amylase gene IDs = contig_14: *FUN_004371*, contig_5: *FUN_008670*, and contig_7 = *FUN_010081*). The alpha-amylase locus on *A. oryzae* 14160 contig_14 displayed conserved synteny with the *A. oryzae* RIB 40 chromosome 2 alpha-amylase locus (Figure 3A). In addition to the chromosome 2 locus, *A. oryzae* RIB 40 harbors alpha-amylase loci on chromosomes 3 and 5, however, we did not observe shared synteny between these loci in *A. oryzae* 14160 (Figures 3B,C).

The *A. oryzae* 14160 genome contained alpha-amylase loci on contig_5 and contig_7, which map to *A. oryzae* RIB40 chromosomes 2 and 6, respectively (Figure 2). The contig_5 locus contains 9 predicted genes not present on the *A. oryzae* RIB 40 genome nested within two syntenic genes (*FUN_008665* and *FUN_008675*) (Figure 3B). Importantly, this locus was assembled identically in the *A. oryzae* 14160 MaSuRCA and Canu assemblies, while the SPAdes assembly was also identical with the exception of an assembly break that did not include an additional copy of the genes *FUN_008673* and *FUN_008668* (Figure 3B). Additionally, we identified several long ONT reads (>30 Kb) that spanned the alpha-amylase gene as well as up-stream and/or down-stream flanking region genes. Specifically, one ONT read (ONT read ID 07fba920-71fc-460e-ba46-46ebda40194a) spanned *FUN_008668*–*FUN_008672* (contig_5:1,224,311–1,258,020) while another (ONT read ID a63546c6-6767-4fcf-9051-9393777f6572) spanned *FUN_008669*–*FUN_008673* (contig_5: 1,231,983–1,265,735). We also identified a ∼9 Kb region nearly identical to the Tc1/mariner class transposable element directly upstream of *FUN_008671* (contig_5: 1,240,697–1,245,364), which has been previously observed in the some alpha-amylase loci in *A. oryzae* strains (Hunter et al., 2011).

The *A. oryzae* 14160 alpha-amylase locus on contig_7 mapped to *A. oryzae* RIB 40 chromosome 6, and displayed conserved synteny for the majority of the locus albeit without the alpha-amylase encoding gene (*FUN_010081*) and the upstream flanking gene (*FUN_010082*) in *A. oryzae* RIB 40 (Figure 3C). We identified two long ONT reads that spanned the genomic regions harboring *FUN_010076*–*FUN_010082* and *FUN_010076*–*FUN_010083*, respectively (ONT read ID 05e9d1bc-f5c1-4a64-85a2-6d5df71f58db = contig_7: 977,243–1,011,315 and ONT read ID f5434ebf-bbda-48dd-8f74-28ed565a5c6b = contig_7: 977,137–1,018,308). Again, we identified the Tc1/mariner class transposable element directly upstream of *FUN_010082* (contig_7: 1,013,761–1,018,433). These results suggest convergent evolution of alpha-amylase duplication in the *A. oryzae* 14160 and RIB 40 genomes.

### Alpha-Amylase Upstream Region Conservation

To investigate if differences in the regulatory region of the alpha-amylase genes corresponded to differences in amylase activity or starch metabolism, we aligned the 1 Kb upstream region of the six alpha-amylase genes. We observed only two polymorphic sites (Supplementary Figure 4). Specifically, we observed a transversion from A to C at one position in the *A. oryzae* 14160 alpha-amylase gene on chromosome 6 (contig_7). In another position, we observed a transversion from T to A in the *A. oryzae* RIB 40 chromosome 2 copy. These results suggest that it is unlikely that differences in the regulatory regions of the alpha-amylase genes contribute to differences in amylase activity or starch metabolism.

### Single Nucleotide Polymorphism Analysis

We used freebayes and vcftools to identify high quality SNPs in *A. oryzae* 14160 relative to the *A. oryzae* RIB 40 reference genome. We identified 130,311 SNPs in *A. oryzae* 14160 (∼1 SNPs per 290 bp) and used SnpEff to annotate and predict the putative impact of these SNPs (Cingolani et al., 2012). ∼35% of SNPs were located within the coding region of genes. Of this subset, 55.64% were silent variants, 43.78% were missense variants, and 0.58% were nonsense variants. We quantified the missense variant rate in each gene which ranged from 0 (no missense variants) to 0.039 (mean = 0.0014, median = 0.00047). We considered the upper 0.05% of per-gene missense variant rates as significant (≥0.0152), which included 60 genes (Supplementary Figure 5). This subset of genes showed no significant enrichment for GO terms. A variety of PFAM protein domains were identified in this subset of genes including transporter, protein kinase, glycosyl hydrolase, endonuclease, transposase, and transcription factor domains (Supplementary Table 1). Additionally, five genes with elevated missense variant rates were part of secondary metabolite encoding gene clusters (*AO090026000589*, *AO090102000459*, *AO090103000221*, *AO090103000351*, and *AO090701000565*), although we did not observe an overrepresentation of genes in secondary metabolite encoding gene clusters compared to the background (Fisher’s Exact Test, *p* = 0.60). Two of the genes with elevated missense variant rates neighbored one another (*AO090003001358* and *AO090003001359*) and both genes encode proteins with predicted glycosyl hydrolases family 18 PFAM domains. Interestingly, the set of genes with elevated missense variant rates had significantly shorter coding sequences compared with the background genes (mean_elevated missense variant rate genes_ = 785 bp, mean_background genes_ = 1,352 bp; Wilcoxon Signed-Rank Test, *p* = 1.1e-9).

Additionally, we examined gene enrichment analysis for the 480 genes that had one or more predicted *HIGH* impact mutations, as defined by SnpEff (i.e., loss of stop codon, gain of stop codon, loss of start codon, splice donor variant, and splice acceptor variant) (Supplementary Table 2). We identified 10 biological process GO terms that were enriched in the genes containing *HIGH* impact mutations [GO:1900557 (emericellamide biosynthetic process), *p* = 0.0003; GO:0050763 (depsipeptide biosynthetic process), *p* = 0.0006; GO:1900555 (emericellamide metabolic process), *p* = 0.0006; GO:0032774 (RNA biosynthetic process), *p* = 0.0013; GO:1901336 (lactone biosynthetic process), *p* = 0.0013; GO:1900560 (austinol biosynthetic process), *p* = 0.0028; GO:1900558 (austinol metabolic process), *p* = 0.0028; GO:1900561 (dehydroaustinol metabolic process), *p* = 0.0036; GO:1900563 (dehydroaustinol biosynthetic process), *p* = 0.0036; GO:0008610 (lipid biosynthetic process), *p* = 0.0062]. In support of the functional enrichment results, we identified *HIGH* impact mutations in 9 of the 75 secondary metabolite biosynthetic “backbone” genes [i.e., polyketide synthase (PKS), non-ribosomal peptide synthetase (NRPS), polyketide synthase/non-ribosomal peptide synthetase hybrid (PKS-NRPS), dimethylallyl tryptophan synthase (DMATS), and diterpene synthase (DTS)]. Of these genes, eight contained nonsense variants (*AO090009000052, AO090009000131, AO090010000404, AO090011000328, AO090038000098, AO090038000543, AO090103000224*, and *AO090103000355*) and one gene contained a nonstop variant (*AO090001000009*). *AO090009000131* and *AO090010000404* contained two nonsense variants, and *AO090011000328* contained a nonsense variant and a splice acceptor variant. Gene length (combined exon length) was not significantly different between genes with *HIGH* impact mutations and genes lacking *HIGH* impact mutations (mean_high impact variant genes_ = 1,418 bp, mean_background genes_ = 1,346 bp; Wilcoxon Signed-Rank Test, *p* = 0.46).

### Lineage Specific Genes

**FIGURE 4:**
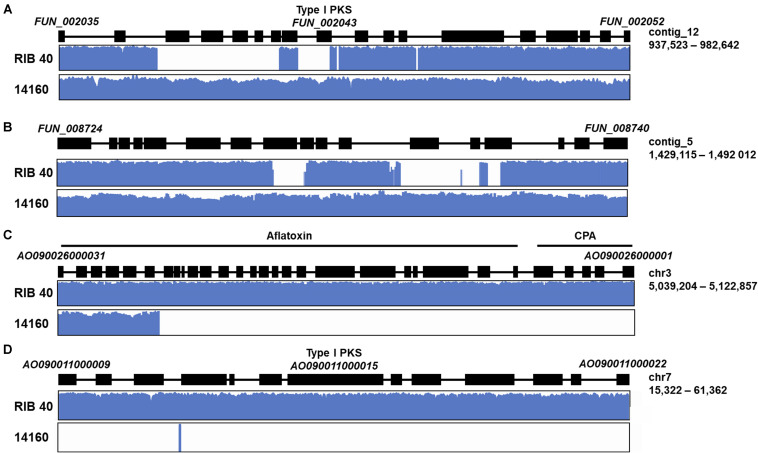
Examples of lineage specific genes in secondary metabolite encoding gene clusters. Lineage specific genes with functional involvement in secondary metabolite production, regulation, and transport in *A. oryzae* 14160 **(A,B)**, and RIB 40 **(C,D)**. For each example, a schematic of the secondary metabolite encoding gene cluster is shown where black boxes represent genes. The first and last gene, scaffold/chromosome ID and scaffold/chromosome coordinates are labeled for each example. Graphs below the gene cluster schematic display the log10 read depth across the gene cluster for each isolate.

We used control-FREEC to predict gene deletions each gene in the *A. oryzae* RIB 40 and *A. oryzae* 14160 reference genomes. Genes that were absent (copy number = 0) in the mapped genome were considered lineage specific genes in the reference genome. Using this approach, we identified 447 and 251 genes in the *A. oryzae* RIB 40 and *A. oryzae* 14160 genomes, respectively (Figure 2 and Supplementary Tables 3, 4). Lineage specific genes were often found in clusters of neighboring genes, likely because of deletion, duplication or insertion events spanning multiple genes. For instance, *A. oryzae* RIB 40 lineage specific genes were found in 87 loci, with only 18 loci containing one gene (average = 5.1, median = 3, max = 34). The largest cluster of lineage specific genes in *A. oryzae* RIB 40 contained 34 genes and overlapped the aflatoxin and cyclopiazonic acid encoding gene clusters (Figure 4C). *A. oryzae* 14160 lineage specific genes were found in 101 loci, with 41 loci containing one gene (average = 2.5, median = 2, max = 14). The largest lineage specific gene cluster in *A. oryzae* 14160 contained 14 genes (*FUN_008412*–*FUN_008425*).

*A. oryzae* RIB 40 lineage specific genes were functionally enriched for the biological process GO terms “secondary metabolite biosynthetic process” (*p* = 7.87e-10), and “sterigmatocystin biosynthetic process” (*p* = 1.35e-5), and the molecular function GO terms “oxidoreductase activity, acting on paired donors, with incorporation or reduction of molecular oxygen” (*p* = 1e-6), “heme binding” (*p* = 1.24e-5), “iron ion binding” (*p* = 1.91e-5), and “electron carrier activity” (*p* = 2.21e-5). Because we observed an overrepresentation of lineage specific genes involved in secondary metabolism in *A. oryzae* RIB 40, we also tested whether this trend was present in *A. oryzae* 14160 lineage specific genes. For this analysis, we tested whether genes annotated within secondary metabolite encoding gene clusters (as annotated by antiSMASH; Medema et al., 2011) were overrepresented in the lineage specific genes vs. the non-lineage specific genes. Indeed, we observed an overrepresentation of genes involved in secondary metabolism in the *A. oryzae* 14160 lineage specific genes (Fisher’s exact test, *p* = 0.0013). Specifically, 11.5% of *A. oryzae* 14160 lineage specific genes were annotated within secondary metabolite encoding gene clusters compared to 5.6% in the background, and these genes fell within 11 independent secondary metabolite encoding gene clusters. Using the same approach, we again identified an enrichment of genes in secondary metabolite encoding gene clusters in *A. oryzae* RIB 40 (Fisher’s exact test, *p* = 2e-11).

### *A. oryzae* 14160 and *A. oryzae* RIB 40 Amylase Activity, Proteolytic Activity, and Growth Rate

**FIGURE 5:**
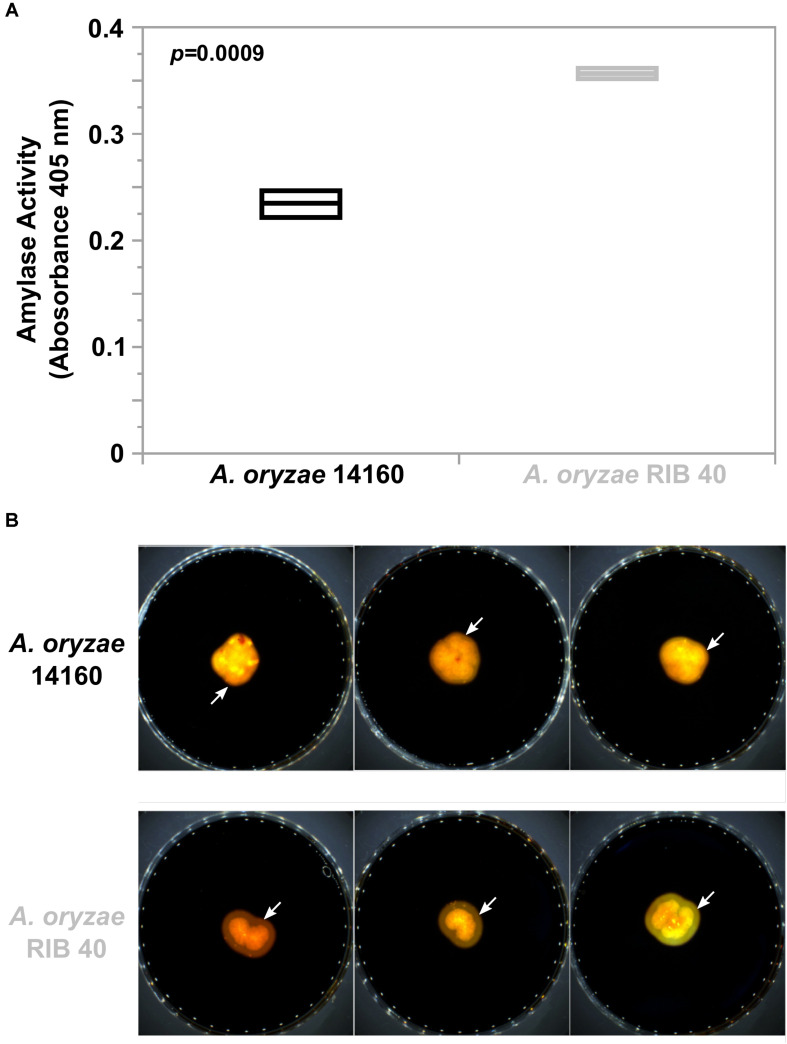
Alpha-amylase activity in *A. oryzae* 14160 and RIB 40. **(A)** Quantitative analysis of amylase activity during solid state rice fermentation. Amylase activity was measured in triplicate using the Megazyme alpha-amylase Assay Kit. Black and Gray box plots and text represent the *A. oryzae* 14160 and RIB 40 isolates, respectively. **(B)** Qualitative iodine based amylase activity assay. Isolates were grown on starch agar and media was stained with iodine. Yellow zones surrounding the fungal colonies (white arrows) indicate amylase activity.

Because we observed widespread genomic variation between *A. oryzae* 14160 and RIB 40, we were interested in how this variation may affect phenotype. Thus, we measured and compared amylase activity and growth rate of both strains. We hypothesized that alpha-amylase activity would be similar between *A. oryzae* 14160 and RIB 40 because both strains possess three copies of the alpha-amylase encoding gene (Figure 3). Interestingly, quantitative analysis of amylase activity during solid-state rice fermentation, and qualitative amylase activity on starch agar showed that *A. oryzae* RIB 40 produces higher levels of amylase (Figure 5). However, *A. oryzae* 14160 and RIB 40 did not significantly differ in their proteolytic activity (Supplementary Figure 6).

**FIGURE 6:**
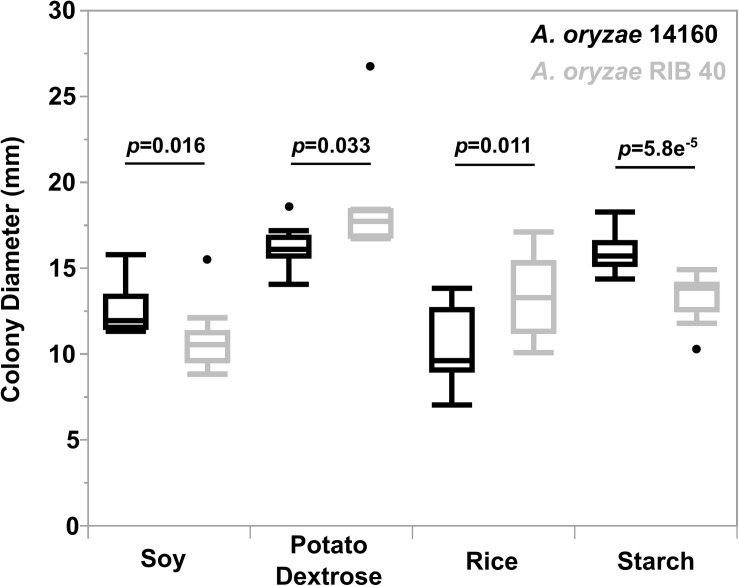
Growth characteristics of *A. oryzae* 14160 and RIB 40 in various media. The Y-axis represents colony growth, while the X-axis represents culture media. Points outside of the boxplots represent outliers. Black and gray boxes represent *A*. *oryzae* 14160 and RIB 40, respectively. *T*-test *p*-values are provided for each culture media.

Both strains showed significantly different growth rates between media types (*A. oryzae* 14160: Oneway Anova, *d.f.* = 3, F-ratio = 31.4, *p* = 1.73e-10 and *A. oryzae* RIB 40: Oneway Anova, *d.f.* = 3, F-ratio = 22.8, *p* = 1.07e-8) (Supplementary Figure 7). *A. oryzae* 14160 grew fastest on PDA and starch, while growing significantly slower on soy, followed by rice (Supplementary Figure 7A). *A. oryzae* RIB 40 grew fastest on PDA, while growth on starch, rice and soy were not significantly different from one another (Supplementary Figure 7B). We also compared the growth rate of *A. oryzae* 14160 vs. RIB 40 for each media type. *A. oryzae* 14160 grew significantly faster on soy (*t*-test, t-ratio = −2.3, *p* = 0.03) and starch (*t*-test, t-ratio = −5.1, *p* = 0.00003) while *A. oryzae* RIB 40 grew significantly faster on PDA (*t*-test, t-ratio = 2.3, *p* = 0.017) and rice (*t*-test, t-ratio = 2.8, *p* = 0.006) (Figure 6).

## Discussion

Here, we used long-read ONT and short-read Illumina sequencing data to assemble an accurate and highly contiguous genome of *A. oryzae* 14160. To date, only four *A. oryzae* isolates have genome assemblies comprised of fewer than 30 scaffolds (RIB40, BP2-1, BCC7051, and TK-29) (Thammarongtham et al., 2018; Watarai et al., 2019; Jeon et al., 2020). These isolates belong to clade F (RIB 40), clade E (BCC7051), clade BP2-1 (BP2-1), and a smaller clade closely related the BP2-1 clade (TK-29) (Watarai et al., 2019). Our phylogenetic analysis revealed that *A. oryzae* 14160 is part of clade A, and thus represents the first highly contiguous genome assembly from this group. Importantly, high quality genome assemblies from representative isolates across clades will enable comparative genomic analysis of structural variants, as we have demonstrated here with our synteny analysis of the alpha-amylase loci (Figure 3).

The assembly and annotation of the *A. oryzae* 14160 genome enabled in depth comparative genomic analysis with the complete chromosome assembly of the *A. oryzae* RIB 40 reference genome. We conducted several analyses to identify genes with divergent patterns (i.e., relative abundance of missense variants, putative impact of variants, and gene presence/absence) in the *A. oryzae* 14160 and RIB 40 genomes. Collectively, these analyses revealed that genes with involvement in secondary metabolism were highly variable (Figures 2, 4). For instance, we observed a large-scale deletion event of the aflatoxin biosynthetic gene cluster that includes more than half of the cluster and the neighboring cyclopiazonic acid encoding gene cluster (Figure 4C). Large-scale chromosomal deletions of the aflatoxin encoding gene cluster have been previously characterized in *A. oryzae* isolates (Lee et al., 2006; Tominaga et al., 2006; Chang and Ehrlich, 2010; Alshannaq et al., 2018). Interestingly, a number of independent loss of function variants have also been observed in *A. oryzae* strains resulting in their inability to produce aflatoxin. This observation indicates the loss of aflatoxin has independently evolved in different *A. oryzae* clades, perhaps to reallocate the high energy demands required to produce this secondary metabolite into primary metabolism (Gibbons, 2019).

In addition we observed a number of high impact variants and gene presence polymorphisms in several putative secondary metabolite backbone encoding genes whose products are not as well-characterized as aflatoxin and cyclopiazonic acid (Figure 4). For example, *FUN_002043* encodes a type I iterative polyketide synthetase and is absent in the *A. oryzae* RIB 40 genome, and a large secondary metabolite encoding gene cluster on *A. oryzae* chromosome 7 is entirely absent from the *A. oryzae* 14160 genome (Figure 4A). These results are consistent with observations in *Aspergillus* species that show secondary metabolite encoding gene clusters are highly variable both between and within species (Gibbons et al., 2012; Ehrlich and Mack, 2014; Lind et al., 2017; Alshannaq et al., 2018; Zhao and Gibbons, 2018; Drott et al., 2020; Kjaerbolling et al., 2020; Steenwyk et al., 2020). For example, genomic analysis of three *Aspergillus nidulans* genomes revealed more than 70 secondary metabolite encoding gene clusters in each genome while nine of these clusters displayed presence/absence polymorphisms (Drott et al., 2020). Similarly, we previously observed a polymorphic locus with two distinct secondary metabolite gene clusters in *A. oryzae* and *A. flavus* (Gibbons et al., 2012).

Alpha-amylase is an enzyme that hydrolyzes the alpha-D-glyosidic bond in starch to produce dextrin, and the high production of this carbohydrate metabolizing enzyme is, perhaps, *A. oryzae*’s most important industrial characteristic. Alpha-amylase copy number varies from one to four in *A. oryzae* isolates and these gene duplication events likely derive from the Tc1/mariner like transposable element that flanks this locus (Hunter et al., 2011; Watarai et al., 2019). Hunter et al. (2011) provided evidence for at least three independent duplication events of the alpha-amylase locus from the ancestral chromosome 2 copy. Interestingly, we also observed conservation of the alpha-amylase locus on chromosome 2 in *A. oryzae* 14160 (Figure 3), which is also conserved in the *A. flavus* NRRL 3357 genome (Nierman et al., 2015). However, we did not observe alpha-amylase copies on chromosomes 3 and 5 as in the *A. oryzae* RIB 40 genome. Instead, we identified an additional copy of the alpha-amylase locus on chromosome 2 and chromosome 6, providing further evidence for convergent evolution of alpha-amylase duplication in *A. oryzae*. The independent duplication of alpha-amylase indicates that artificial selection for increased amylase production was very strong during the domestication of *A. oryzae*.

Because we observed extensive genome variation between *A. oryzae* 14160 and RIB 40 we investigated how these strains differed phenotypically. First, we measure amylase activity using two independent assays. Both assays showed that *A. oryzae* RIB 40 produces greater levels of alpha-amylase (Figure 5). This observation was somewhat surprising considering the genomes of both strains contain three copies of the alpha-amylase encoding gene, and the upstream regions of these genes are nearly identical (Supplementary Figure 4). However, a study that generated single, double, and triple disruptant mutants of the three alpha-amylase encoding genes in RIB 40 revealed that the contribution of amylase gene and protein expression was not equal between the three copies (Nemoto et al., 2012). More specifically, *amyA* (the conserved alpha-amylase copy on chromosome 2) contributed least to amylase production. Consequently, the newly duplicated copies of alpha-amylase may also dominate amylase expression in other *A. oryzae* isolates, and chromosomal location of alpha-amylase paralogs may influence their gene expression. For instance, position effect variegation was observed in *A. nidulans* where a translocation of the developmental regulator *brlA* resulted a conidiophore that remained as a stiff hyphae and did not develop a vesicle, sterigmata, and conidia (Clutterbuck, 1970). Similarly, the chromosomal position of the alpha-amylase loci in *A. oryzae* could potentially influence expression.

Finally, we observed differential growth preferences between *A. oryzae* 14160 and RIB 40. Interestingly, *A. oryzae* 14160 grew significantly faster on soy and starch agar, while RIB 40 grew significantly faster on PDA and rice agar. Though *A. oryzae* RIB 40 did not grow faster on starch agar, amylase activity was visibly greater, suggesting that growth rate may have increased during a longer incubation period. Additionally, the starch agar contained beef extract which provides a source of proteins and peptides. Thus, *A. oryzae* 14160 grew faster where protein content was higher (soy and starch), and RIB 40 grew faster when carbohydrates were the major available energy source (potato dextrose and rice). This observation suggests that *A. oryzae* 14160 is better suited for soy fermentation, while RIB 40 is better suited for rice fermentation.

## Data Availability Statement

The datasets presented in this study can be found in online repositories. The names of the repository/repositories and accession number(s) can be found below: https://www.ncbi.nlm.nih.gov/, PRJNA717291.

## Author Contributions

KC-V and JG conducted genomic and statistical analysis. CM performed growth rate and amylase experiments. J-HY and DC maintained the cultures and extracted DNA for Illumina sequencing. LW provided the *A. oryzae* 14160. All authors contributed to manuscript revision and approved the submitted version.

## Conflict of Interest

The authors declare that the research was conducted in the absence of any commercial or financial relationships that could be construed as a potential conflict of interest.

## Footnotes

1 http://www.bioinformatics.babraham.ac.uk/projects/trim_galore/

2 https://www.bioinformatics.babraham.ac.uk/projects/fastqc/

3 https://github.com/nextgenusfs/funannotate

4 https://elbe.hki-jena.de/fungifun/
