# Supplementary material for: Comparison of Two Aspergillus oryzae Genomes From Different Clades Reveals Independent Evolution of Alpha-Amylase Duplication, Variation in Secondary Metabolism Genes, and Differences in Primary Metabolism
Source: Front Microbiol. 2021 Jul 13;12:691296. doi: 10.3389/fmicb.2021.691296 (PMC8313989; doi:10.3389/fmicb.2021.691296)
Supplement: Supplementary file 1 [file Data_Sheet_1.zip › Table 1 (1).DOCX]

**Supplementary Table S1. PFAM domains in proteins with elevated missense variant rate (≥0.0152) between *A. oryzae* 14160 and RIB 40.**

| **Gene Name** | **PFAM Domain** | **PFAM Identifier** | **E-value** | **Description** |
| --- | --- | --- | --- | --- |
| AO090001000129 | DUF3632 | PF12311.8 | 9.8E-39 | Protein of unknown function (DUF3632) |
| AO090001000155 | AUDH_Cupin | PF18637.1 | 3.9E-64 | Aldos-2-ulose dehydratase/isomerase (AUDH) Cupin domain |
| AO090001000155 | PBP3 | PF18056.1 | 6.7E-24 | Penicillin Binding Protein 3 Domain |
| AO090003001358 | Glyco_hydro_18 | PF00704.28 | 2.3E-12 | Glycosyl hydrolases family 18 |
| AO090003001359 | Glyco_hydro_18 | PF00704.28 | 1.8E-27 | Glycosyl hydrolases family 18 |
| AO090003001399 | MFS_1 | PF07690.16 | 3.9E-29 | Major Facilitator Superfamily |
| AO090003001426 | Abhydrolase_6 | PF12697.7 | 1.3E-18 | Alpha/beta hydrolase family |
| AO090005000381 | HET | PF06985.11 | 1.1E-31 | Heterokaryon incompatibility protein (HET) |
| AO090010000385 | Pkinase | PF00069.25 | 3.7E-36 | Protein kinase domain |
| AO090010000568 | WD40 | PF00400.32 | 1.6E-13 | WD domain, G-beta repeat |
| AO090011000234 | DUF346 | PF03984.13 | 3.5E-14 | Repeat of unknown function (DUF346) |
| AO090012000870 | Profilin | PF00235.19 | 3.7E-24 | Profilin |
| AO090020000451 | DDE_1 | PF03184.19 | 7.6E-19 | DDE superfamily endonuclease |
| AO090020000451 | HTH_Tnp_Tc5 | PF03221.16 | 1.4E-13 | Tc5 transposase DNA-binding domain |
| AO090026000313 | ABC_membrane | PF00664.23 | 2.4E-11 | ABC transporter transmembrane region |
| AO090026000313 | ABC_tran | PF00005.27 | 1.4E-31 | ABC transporter |
| AO090102000459 | Fungal_trans_2 | PF11951.8 | 2E-11 | Fungal specific transcription factor domain |
| AO090103000142 | ANAPC4_WD40 | PF12894.7 | 8.8E-11 | Anaphase-promoting complex subunit 4 WD40 domain |
| AO090103000142 | WD40 | PF00400.32 | 4.2E-13 | WD domain, G-beta repeat |
| AO090103000394 | Sugar_tr | PF00083.24 | 4E-24 | Sugar (and other) transporter |
| AO090113000042 | Sugar_tr | PF00083.24 | 9.3E-65 | Sugar (and other) transporter |
| AO090113000066 | TPR_12 | PF13424.6 | 2.2E-18 | Tetratricopeptide repeat |
| AO090206000090 | Ank_2 | PF12796.7 | 3.8E-54 | Ankyrin repeats (3 copies) |
| AO090206000090 | Ank_4 | PF13637.6 | 2.8E-51 | Ankyrin repeats (many copies) |
