# Supplementary material for: Comparison of Two Aspergillus oryzae Genomes From Different Clades Reveals Independent Evolution of Alpha-Amylase Duplication, Variation in Secondary Metabolism Genes, and Differences in Primary Metabolism
Source: Front Microbiol. 2021 Jul 13;12:691296. doi: 10.3389/fmicb.2021.691296 (PMC8313989; doi:10.3389/fmicb.2021.691296)
Supplement: Supplementary file 1 [file Data_Sheet_1.zip › Table 2.DOCX]

**Supplementary Table S2. Genes with SNPeff annotated “HIGH IMPACT” variants between *A. oryzae* 14160 and RIB 40.** The frequency of each high impact variant is provided for each gene.

| Gene Id | splice_acceptor | splice_donor | start_lost | stop_gained | stop_lost |
| --- | --- | --- | --- | --- | --- |
| AO090001000006 | 0 | 0 | 0 | 0 | 1 |
| AO090001000007 | 0 | 0 | 0 | 1 | 0 |
| AO090001000009 | 0 | 0 | 0 | 0 | 1 |
| AO090001000062 | 0 | 0 | 1 | 0 | 0 |
| AO090001000084 | 0 | 1 | 0 | 0 | 0 |
| AO090001000103 | 0 | 0 | 0 | 1 | 0 |
| AO090001000106 | 1 | 1 | 0 | 0 | 0 |
| AO090001000117 | 0 | 0 | 0 | 1 | 0 |
| AO090001000133 | 0 | 0 | 1 | 0 | 0 |
| AO090001000149 | 0 | 0 | 0 | 2 | 1 |
| AO090001000182 | 0 | 1 | 0 | 0 | 0 |
| AO090001000183 | 1 | 0 | 0 | 0 | 0 |
| AO090001000211 | 0 | 0 | 0 | 0 | 1 |
| AO090001000244 | 0 | 0 | 1 | 0 | 0 |
| AO090001000246 | 0 | 0 | 0 | 1 | 0 |
| AO090001000254 | 0 | 0 | 0 | 1 | 0 |
| AO090001000259 | 0 | 0 | 1 | 0 | 0 |
| AO090001000263 | 0 | 0 | 0 | 0 | 1 |
| AO090001000267 | 0 | 0 | 0 | 1 | 0 |
| AO090001000270 | 1 | 0 | 0 | 0 | 0 |
| AO090001000381 | 0 | 1 | 0 | 0 | 0 |
| AO090001000407 | 0 | 0 | 0 | 1 | 0 |
| AO090001000477 | 0 | 0 | 0 | 1 | 0 |
| AO090001000486 | 0 | 0 | 0 | 1 | 0 |
| AO090001000489 | 0 | 0 | 0 | 1 | 0 |
| AO090001000534 | 0 | 0 | 1 | 1 | 0 |
| AO090001000540 | 0 | 0 | 0 | 1 | 0 |
| AO090001000542 | 0 | 0 | 0 | 1 | 0 |
| AO090001000588 | 0 | 0 | 0 | 1 | 0 |
| AO090003000136 | 0 | 0 | 0 | 0 | 1 |
| AO090003000194 | 0 | 0 | 0 | 1 | 0 |
| AO090003000241 | 0 | 0 | 0 | 1 | 0 |
| AO090003000335 | 0 | 0 | 0 | 0 | 1 |
| AO090003000357 | 0 | 0 | 0 | 1 | 0 |
| AO090003000361 | 0 | 1 | 0 | 0 | 0 |
| AO090003000379 | 0 | 0 | 1 | 0 | 0 |
| AO090003000527 | 0 | 0 | 0 | 0 | 1 |
| AO090003000562 | 0 | 1 | 0 | 0 | 0 |
| AO090003000601 | 0 | 0 | 0 | 1 | 0 |
| AO090003000614 | 1 | 0 | 0 | 0 | 0 |
| AO090003000694 | 1 | 0 | 0 | 0 | 0 |
| AO090003000703 | 0 | 1 | 0 | 0 | 0 |
| AO090003000734 | 0 | 0 | 0 | 1 | 0 |
| AO090003000787 | 0 | 0 | 0 | 1 | 0 |
| AO090003000870 | 0 | 0 | 0 | 0 | 1 |
| AO090003000932 | 0 | 0 | 0 | 0 | 1 |
| AO090003000984 | 0 | 0 | 0 | 0 | 1 |
| AO090003001014 | 0 | 0 | 0 | 1 | 0 |
| AO090003001208 | 0 | 0 | 1 | 0 | 0 |
| AO090003001286 | 0 | 0 | 1 | 0 | 0 |
| AO090003001303 | 0 | 0 | 0 | 1 | 0 |
| AO090003001308 | 1 | 0 | 0 | 0 | 0 |
| AO090003001359 | 0 | 0 | 0 | 0 | 1 |
| AO090003001399 | 0 | 0 | 0 | 1 | 0 |
| AO090003001425 | 0 | 0 | 0 | 1 | 0 |
| AO090001t00004 | 0 | 0 | 0 | 0 | 1 |
| AO090003001468 | 2 | 1 | 0 | 0 | 0 |
| AO090003001469 | 0 | 0 | 0 | 1 | 0 |
| AO090003001481 | 1 | 0 | 0 | 0 | 0 |
| AO090003001517 | 0 | 0 | 1 | 0 | 0 |
| AO090005000003 | 0 | 0 | 0 | 1 | 0 |
| AO090005000007 | 0 | 0 | 0 | 1 | 0 |
| AO090005000052 | 0 | 0 | 1 | 0 | 1 |
| AO090005000133 | 0 | 0 | 0 | 1 | 0 |
| AO090005000150 | 0 | 0 | 0 | 1 | 0 |
| AO090005000167 | 0 | 0 | 0 | 1 | 0 |
| AO090005000179 | 0 | 0 | 0 | 0 | 1 |
| AO090005000194 | 0 | 0 | 0 | 1 | 0 |
| AO090005000196 | 0 | 0 | 0 | 1 | 0 |
| AO090005000201 | 0 | 0 | 0 | 0 | 1 |
| AO090005000207 | 1 | 0 | 0 | 0 | 0 |
| AO090005000230 | 0 | 0 | 0 | 1 | 0 |
| AO090005000234 | 1 | 0 | 0 | 0 | 1 |
| AO090005000240 | 0 | 0 | 0 | 1 | 0 |
| AO090005000262 | 0 | 0 | 0 | 1 | 0 |
| AO090005000272 | 0 | 0 | 1 | 0 | 0 |
| AO090005000294 | 0 | 0 | 0 | 0 | 1 |
| AO090005000359 | 0 | 0 | 1 | 0 | 0 |
| AO090005000362 | 0 | 0 | 0 | 1 | 0 |
| AO090005000378 | 0 | 0 | 0 | 1 | 0 |
| AO090005000382 | 0 | 1 | 1 | 0 | 0 |
| AO090005000388 | 0 | 0 | 0 | 0 | 1 |
| AO090005000389 | 0 | 0 | 0 | 1 | 0 |
| AO090005000391 | 0 | 0 | 0 | 0 | 1 |
| AO090005000402 | 0 | 0 | 0 | 1 | 0 |
| AO090005000403 | 0 | 0 | 0 | 3 | 0 |
| AO090005000448 | 0 | 0 | 0 | 1 | 0 |
| AO090005000485 | 0 | 1 | 0 | 0 | 0 |
| AO090005000501 | 0 | 0 | 0 | 1 | 0 |
| AO090005000507 | 0 | 0 | 0 | 0 | 1 |
| AO090005000512 | 0 | 0 | 0 | 0 | 1 |
| AO090005000533 | 1 | 0 | 0 | 0 | 0 |
| AO090005000580 | 0 | 0 | 0 | 0 | 1 |
| AO090005000649 | 1 | 0 | 0 | 0 | 0 |
| AO090005000662 | 0 | 0 | 0 | 1 | 0 |
| AO090005000671 | 0 | 0 | 0 | 0 | 1 |
| AO090005000685 | 0 | 0 | 0 | 1 | 0 |
| AO090005000809 | 0 | 0 | 0 | 0 | 1 |
| AO090005000843 | 0 | 1 | 0 | 0 | 0 |
| AO090005000869 | 0 | 0 | 0 | 0 | 1 |
| AO090005000902 | 0 | 1 | 0 | 0 | 0 |
| AO090005000983 | 0 | 0 | 0 | 1 | 0 |
| AO090005000984 | 0 | 0 | 1 | 0 | 0 |
| AO090005000991 | 0 | 1 | 0 | 0 | 0 |
| AO090005001020 | 1 | 0 | 0 | 0 | 0 |
| AO090005001032 | 0 | 0 | 0 | 1 | 0 |
| AO090005001081 | 1 | 0 | 0 | 0 | 0 |
| AO090005001149 | 1 | 0 | 0 | 0 | 0 |
| AO090005001174 | 0 | 0 | 0 | 1 | 0 |
| AO090005001183 | 0 | 1 | 0 | 0 | 0 |
| AO090005001191 | 0 | 1 | 0 | 0 | 0 |
| AO090005001368 | 0 | 0 | 0 | 0 | 1 |
| AO090005001377 | 1 | 1 | 0 | 1 | 0 |
| AO090005001560 | 0 | 0 | 0 | 1 | 0 |
| AO090005001563 | 0 | 0 | 0 | 0 | 1 |
| AO090005001566 | 1 | 0 | 0 | 0 | 0 |
| AO090009000028 | 0 | 0 | 0 | 1 | 0 |
| AO090009000035 | 0 | 0 | 0 | 1 | 0 |
| AO090009000040 | 0 | 1 | 0 | 0 | 0 |
| AO090009000047 | 0 | 0 | 0 | 1 | 0 |
| AO090009000052 | 0 | 0 | 0 | 1 | 0 |
| AO090009000056 | 0 | 0 | 0 | 0 | 1 |
| AO090009000070 | 0 | 0 | 0 | 0 | 1 |
| AO090009000076 | 0 | 0 | 1 | 0 | 0 |
| AO090009000106 | 0 | 0 | 0 | 1 | 0 |
| AO090009000107 | 0 | 0 | 0 | 1 | 0 |
| AO090009000131 | 0 | 0 | 0 | 2 | 0 |
| AO090009000241 | 0 | 0 | 0 | 0 | 1 |
| AO090009000280 | 0 | 0 | 0 | 1 | 0 |
| AO090009000373 | 0 | 0 | 0 | 1 | 0 |
| AO090009000406 | 1 | 0 | 0 | 0 | 0 |
| AO090009000470 | 0 | 1 | 0 | 0 | 0 |
| AO090009000509 | 0 | 0 | 0 | 1 | 1 |
| AO090009000511 | 0 | 0 | 0 | 1 | 0 |
| AO090009000522 | 0 | 1 | 0 | 0 | 0 |
| AO090009000523 | 0 | 0 | 1 | 0 | 0 |
| AO090009000539 | 0 | 0 | 0 | 1 | 1 |
| AO090009000551 | 0 | 0 | 0 | 0 | 1 |
| AO090009000572 | 0 | 0 | 0 | 1 | 0 |
| AO090009000576 | 0 | 0 | 0 | 0 | 1 |
| AO090009000602 | 0 | 0 | 0 | 1 | 0 |
| AO090009000619 | 0 | 1 | 0 | 0 | 0 |
| AO090010000009 | 0 | 0 | 0 | 0 | 1 |
| AO090010000061 | 0 | 0 | 0 | 0 | 1 |
| AO090010000068 | 0 | 0 | 0 | 0 | 1 |
| AO090010000070 | 0 | 0 | 0 | 1 | 0 |
| AO090010000080 | 0 | 1 | 0 | 0 | 0 |
| AO090010000112 | 0 | 0 | 0 | 1 | 0 |
| AO090010000130 | 1 | 0 | 0 | 0 | 0 |
| AO090010000143 | 0 | 0 | 1 | 0 | 0 |
| AO090010000183 | 0 | 0 | 1 | 0 | 0 |
| AO090010000185 | 0 | 0 | 0 | 1 | 0 |
| AO090010000193 | 0 | 0 | 0 | 1 | 0 |
| AO090010000215 | 1 | 0 | 0 | 0 | 0 |
| AO090010000220 | 0 | 0 | 1 | 0 | 0 |
| AO090010000251 | 0 | 0 | 0 | 1 | 0 |
| AO090010000270 | 0 | 0 | 0 | 1 | 0 |
| AO090010000320 | 1 | 0 | 0 | 0 | 0 |
| AO090010000346 | 0 | 0 | 0 | 1 | 0 |
| AO090010000350 | 0 | 1 | 0 | 0 | 0 |
| AO090010000355 | 0 | 0 | 0 | 0 | 1 |
| AO090010000385 | 0 | 0 | 0 | 1 | 0 |
| AO090010000388 | 0 | 1 | 0 | 0 | 0 |
| AO090010000404 | 0 | 0 | 0 | 2 | 0 |
| AO090010000431 | 0 | 0 | 0 | 1 | 0 |
| AO090010000516 | 0 | 0 | 0 | 2 | 0 |
| AO090010000521 | 0 | 0 | 0 | 0 | 1 |
| AO090010000556 | 1 | 0 | 0 | 0 | 0 |
| AO090010000561 | 0 | 0 | 0 | 1 | 0 |
| AO090010000569 | 1 | 0 | 0 | 0 | 0 |
| AO090010000573 | 1 | 0 | 0 | 0 | 0 |
| AO090010000583 | 0 | 0 | 0 | 1 | 0 |
| AO090010000588 | 0 | 0 | 0 | 1 | 0 |
| AO090010000595 | 0 | 0 | 0 | 1 | 0 |
| AO090010000607 | 0 | 0 | 0 | 1 | 0 |
| AO090010000613 | 1 | 0 | 0 | 0 | 0 |
| AO090010000631 | 1 | 0 | 0 | 0 | 0 |
| AO090010000642 | 0 | 0 | 0 | 1 | 0 |
| AO090010000671 | 0 | 0 | 0 | 1 | 0 |
| AO090010000675 | 1 | 0 | 0 | 1 | 0 |
| AO090010000690 | 0 | 0 | 0 | 1 | 0 |
| AO090010000704 | 0 | 0 | 0 | 1 | 0 |
| AO090010000755 | 0 | 1 | 0 | 0 | 0 |
| AO090010000762 | 1 | 0 | 0 | 0 | 0 |
| AO090010000778 | 0 | 0 | 1 | 0 | 0 |
| AO090005t00025 | 0 | 0 | 0 | 1 | 0 |
| AO090009t00007 | 0 | 0 | 0 | 1 | 0 |
| AO090011000028 | 0 | 1 | 0 | 0 | 0 |
| AO090011000034 | 0 | 0 | 0 | 1 | 0 |
| AO090011000057 | 0 | 0 | 0 | 1 | 0 |
| AO090011000081 | 0 | 0 | 0 | 1 | 0 |
| AO090011000090 | 0 | 0 | 1 | 0 | 0 |
| AO090011000092 | 0 | 0 | 0 | 1 | 0 |
| AO090011000101 | 0 | 0 | 0 | 1 | 0 |
| AO090011000107 | 0 | 0 | 0 | 1 | 0 |
| AO090011000203 | 0 | 0 | 0 | 0 | 1 |
| AO090011000218 | 0 | 0 | 0 | 1 | 0 |
| AO090011000242 | 0 | 1 | 0 | 0 | 0 |
| AO090011000249 | 0 | 0 | 0 | 1 | 0 |
| AO090011000252 | 0 | 0 | 0 | 1 | 0 |
| AO090011000271 | 0 | 0 | 0 | 1 | 0 |
| AO090011000303 | 1 | 0 | 0 | 1 | 0 |
| AO090011000328 | 1 | 0 | 0 | 1 | 0 |
| AO090011000346 | 0 | 0 | 0 | 1 | 0 |
| AO090011000353 | 0 | 1 | 0 | 0 | 0 |
| AO090011000356 | 0 | 0 | 0 | 1 | 0 |
| AO090011000383 | 0 | 0 | 0 | 1 | 0 |
| AO090011000416 | 0 | 0 | 0 | 1 | 0 |
| AO090011000464 | 0 | 0 | 0 | 1 | 0 |
| AO090011000474 | 0 | 0 | 0 | 1 | 0 |
| AO090011000484 | 0 | 0 | 0 | 1 | 0 |
| AO090011000525 | 0 | 0 | 0 | 1 | 1 |
| AO090011000556 | 0 | 0 | 0 | 1 | 0 |
| AO090011000567 | 0 | 0 | 0 | 2 | 0 |
| AO090011000675 | 0 | 0 | 0 | 0 | 1 |
| AO090011000676 | 0 | 0 | 0 | 1 | 0 |
| AO090011000677 | 0 | 1 | 0 | 0 | 0 |
| AO090011000684 | 0 | 0 | 0 | 1 | 0 |
| AO090011000704 | 1 | 0 | 0 | 0 | 0 |
| AO090011000713 | 0 | 0 | 1 | 0 | 0 |
| AO090011000726 | 0 | 0 | 0 | 0 | 1 |
| AO090011000736 | 0 | 0 | 0 | 0 | 1 |
| AO090011000788 | 0 | 0 | 0 | 1 | 0 |
| AO090011000815 | 0 | 0 | 0 | 1 | 0 |
| AO090011000866 | 0 | 0 | 0 | 1 | 0 |
| AO090012000008 | 0 | 0 | 0 | 1 | 0 |
| AO090012000009 | 0 | 0 | 0 | 0 | 1 |
| AO090012000011 | 1 | 0 | 0 | 0 | 0 |
| AO090012000031 | 0 | 0 | 0 | 1 | 0 |
| AO090012000042 | 0 | 0 | 0 | 0 | 1 |
| AO090012000048 | 1 | 0 | 0 | 0 | 0 |
| AO090012000105 | 1 | 0 | 0 | 0 | 0 |
| AO090012000109 | 0 | 0 | 0 | 1 | 0 |
| AO090012000116 | 0 | 0 | 0 | 0 | 1 |
| AO090012000255 | 0 | 0 | 0 | 1 | 0 |
| AO090012000281 | 0 | 0 | 0 | 0 | 1 |
| AO090012000310 | 0 | 0 | 0 | 0 | 1 |
| AO090012000316 | 1 | 0 | 0 | 0 | 0 |
| AO090012000387 | 0 | 0 | 1 | 0 | 0 |
| AO090012000462 | 0 | 0 | 0 | 1 | 0 |
| AO090012000465 | 0 | 0 | 0 | 1 | 0 |
| AO090012000482 | 0 | 0 | 0 | 1 | 0 |
| AO090012000545 | 0 | 0 | 0 | 1 | 0 |
| AO090012000665 | 0 | 0 | 0 | 0 | 1 |
| AO090012000667 | 0 | 0 | 0 | 1 | 0 |
| AO090012000694 | 0 | 0 | 0 | 0 | 1 |
| AO090012000704 | 0 | 0 | 0 | 1 | 0 |
| AO090012000748 | 0 | 0 | 0 | 0 | 1 |
| AO090012000783 | 1 | 0 | 0 | 0 | 0 |
| AO090012000894 | 0 | 0 | 0 | 0 | 1 |
| AO090012000904 | 0 | 0 | 1 | 0 | 0 |
| AO090020000005 | 0 | 0 | 0 | 0 | 1 |
| AO090020000039 | 0 | 0 | 0 | 1 | 0 |
| AO090020000163 | 0 | 0 | 0 | 1 | 0 |
| AO090020000171 | 1 | 0 | 0 | 0 | 0 |
| AO090020000180 | 0 | 1 | 0 | 0 | 0 |
| AO090020000213 | 0 | 0 | 0 | 1 | 0 |
| AO090020000221 | 0 | 1 | 0 | 0 | 0 |
| AO090020000232 | 0 | 0 | 0 | 1 | 0 |
| AO090020000233 | 0 | 0 | 0 | 1 | 0 |
| AO090020000239 | 0 | 0 | 0 | 1 | 0 |
| AO090020000244 | 0 | 0 | 0 | 0 | 1 |
| AO090020000296 | 0 | 0 | 0 | 0 | 1 |
| AO090020000302 | 1 | 0 | 0 | 0 | 0 |
| AO090020000451 | 0 | 0 | 0 | 1 | 0 |
| AO090020000496 | 0 | 1 | 0 | 0 | 0 |
| AO090020000542 | 0 | 0 | 0 | 0 | 1 |
| AO090020000568 | 0 | 0 | 0 | 0 | 1 |
| AO090020000610 | 0 | 0 | 0 | 1 | 0 |
| AO090020000652 | 0 | 0 | 1 | 0 | 0 |
| AO090020000704 | 0 | 0 | 0 | 1 | 0 |
| AO090020000715 | 0 | 0 | 0 | 0 | 1 |
| AO090023000055 | 0 | 0 | 0 | 1 | 0 |
| AO090023000063 | 0 | 0 | 1 | 0 | 0 |
| AO090023000074 | 0 | 0 | 0 | 0 | 1 |
| AO090023000185 | 0 | 0 | 0 | 1 | 0 |
| AO090023000194 | 0 | 0 | 0 | 1 | 0 |
| AO090023000212 | 0 | 0 | 0 | 1 | 0 |
| AO090023000239 | 0 | 0 | 0 | 0 | 1 |
| AO090023000244 | 0 | 0 | 0 | 1 | 0 |
| AO090023000249 | 1 | 0 | 0 | 0 | 0 |
| AO090023000253 | 0 | 0 | 0 | 1 | 0 |
| AO090023000263 | 1 | 0 | 0 | 0 | 0 |
| AO090023000290 | 0 | 0 | 1 | 1 | 0 |
| AO090023000329 | 0 | 0 | 0 | 1 | 0 |
| AO090023000352 | 0 | 0 | 0 | 1 | 0 |
| AO090023000392 | 1 | 0 | 0 | 0 | 0 |
| AO090023000397 | 1 | 0 | 0 | 0 | 0 |
| AO090023000407 | 0 | 0 | 0 | 1 | 0 |
| AO090023000414 | 1 | 0 | 0 | 0 | 0 |
| AO090023000422 | 0 | 0 | 0 | 1 | 0 |
| AO090023000430 | 0 | 0 | 0 | 0 | 1 |
| AO090023000490 | 0 | 0 | 0 | 0 | 1 |
| AO090023000502 | 0 | 0 | 1 | 0 | 0 |
| AO090023000605 | 0 | 1 | 0 | 0 | 0 |
| AO090023000612 | 0 | 0 | 0 | 1 | 0 |
| AO090023000615 | 0 | 0 | 0 | 1 | 0 |
| AO090023000658 | 0 | 0 | 0 | 1 | 0 |
| AO090023000832 | 0 | 1 | 0 | 0 | 0 |
| AO090023000876 | 0 | 1 | 0 | 0 | 0 |
| AO090023t00001 | 0 | 0 | 0 | 0 | 1 |
| AO090023t00003 | 0 | 0 | 0 | 0 | 1 |
| AO090026000088 | 0 | 0 | 0 | 0 | 1 |
| AO090026000117 | 0 | 0 | 0 | 1 | 1 |
| AO090026000118 | 0 | 0 | 0 | 1 | 0 |
| AO090026000179 | 0 | 0 | 0 | 1 | 0 |
| AO090026000187 | 0 | 0 | 0 | 1 | 0 |
| AO090026000209 | 0 | 1 | 0 | 0 | 0 |
| AO090026000251 | 0 | 0 | 0 | 0 | 1 |
| AO090026000304 | 0 | 0 | 0 | 1 | 0 |
| AO090026000312 | 0 | 0 | 0 | 1 | 0 |
| AO090026000313 | 0 | 2 | 0 | 1 | 0 |
| AO090026000564 | 0 | 0 | 0 | 0 | 1 |
| AO090026000567 | 0 | 0 | 0 | 1 | 0 |
| AO090026000573 | 0 | 1 | 0 | 0 | 0 |
| AO090026000629 | 0 | 0 | 0 | 1 | 0 |
| AO090026000660 | 0 | 1 | 0 | 0 | 0 |
| AO090026000762 | 0 | 0 | 0 | 1 | 0 |
| AO090038000020 | 0 | 0 | 1 | 0 | 0 |
| AO090038000026 | 0 | 0 | 0 | 1 | 0 |
| AO090038000033 | 0 | 0 | 1 | 0 | 0 |
| AO090038000062 | 0 | 1 | 0 | 0 | 0 |
| AO090038000098 | 0 | 0 | 0 | 1 | 0 |
| AO090038000118 | 0 | 0 | 0 | 1 | 0 |
| AO090038000132 | 0 | 1 | 0 | 1 | 0 |
| AO090038000153 | 0 | 0 | 0 | 2 | 0 |
| AO090038000162 | 0 | 0 | 0 | 1 | 0 |
| AO090038000187 | 0 | 1 | 0 | 0 | 0 |
| AO090038000195 | 0 | 1 | 0 | 0 | 0 |
| AO090038000199 | 0 | 0 | 0 | 1 | 1 |
| AO090038000292 | 0 | 0 | 1 | 0 | 0 |
| AO090038000422 | 1 | 0 | 0 | 1 | 0 |
| AO090038000439 | 0 | 0 | 0 | 1 | 0 |
| AO090038000457 | 0 | 0 | 0 | 1 | 0 |
| AO090038000473 | 0 | 0 | 0 | 0 | 1 |
| AO090038000505 | 0 | 0 | 0 | 1 | 0 |
| AO090038000543 | 0 | 0 | 0 | 1 | 0 |
| AO090102000017 | 0 | 1 | 0 | 0 | 0 |
| AO090102000018 | 0 | 1 | 0 | 0 | 0 |
| AO090102000033 | 1 | 0 | 0 | 0 | 0 |
| AO090102000051 | 1 | 0 | 0 | 0 | 0 |
| AO090102000078 | 0 | 1 | 0 | 0 | 0 |
| AO090102000180 | 0 | 0 | 0 | 0 | 1 |
| AO090102000199 | 0 | 0 | 0 | 1 | 0 |
| AO090102000201 | 0 | 0 | 0 | 2 | 0 |
| AO090102000203 | 0 | 0 | 0 | 0 | 1 |
| AO090102000217 | 0 | 0 | 0 | 0 | 1 |
| AO090102000256 | 0 | 0 | 0 | 1 | 0 |
| AO090102000269 | 0 | 1 | 0 | 0 | 0 |
| AO090102000286 | 1 | 0 | 0 | 1 | 0 |
| AO090102000304 | 1 | 0 | 0 | 0 | 0 |
| AO090102000306 | 0 | 0 | 0 | 0 | 1 |
| AO090102000307 | 0 | 0 | 1 | 0 | 0 |
| AO090102000351 | 0 | 1 | 0 | 0 | 0 |
| AO090102000405 | 0 | 0 | 0 | 1 | 0 |
| AO090102000410 | 0 | 0 | 0 | 1 | 0 |
| AO090102000411 | 1 | 0 | 0 | 0 | 0 |
| AO090102000448 | 1 | 0 | 0 | 0 | 0 |
| AO090102000449 | 0 | 0 | 0 | 1 | 0 |
| AO090102000450 | 0 | 1 | 0 | 0 | 0 |
| AO090102000469 | 0 | 0 | 0 | 2 | 0 |
| AO090102000576 | 0 | 0 | 0 | 1 | 0 |
| AO090102000642 | 0 | 0 | 1 | 0 | 0 |
| AO090102000649 | 0 | 1 | 0 | 0 | 0 |
| AO090103000096 | 0 | 0 | 0 | 1 | 0 |
| AO090103000114 | 0 | 0 | 0 | 1 | 0 |
| AO090103000115 | 0 | 0 | 0 | 1 | 0 |
| AO090103000128 | 0 | 0 | 0 | 0 | 1 |
| AO090103000146 | 1 | 0 | 0 | 1 | 0 |
| AO090103000152 | 0 | 0 | 0 | 1 | 0 |
| AO090103000153 | 0 | 1 | 0 | 0 | 0 |
| AO090103000176 | 0 | 0 | 0 | 1 | 0 |
| AO090103000180 | 0 | 0 | 0 | 1 | 0 |
| AO090103000193 | 0 | 1 | 0 | 0 | 1 |
| AO090103000200 | 0 | 0 | 1 | 0 | 1 |
| AO090103000214 | 0 | 0 | 0 | 1 | 0 |
| AO090103000217 | 0 | 1 | 0 | 0 | 0 |
| AO090103000224 | 0 | 0 | 0 | 1 | 0 |
| AO090103000226 | 0 | 0 | 0 | 1 | 0 |
| AO090103000246 | 0 | 0 | 1 | 0 | 0 |
| AO090103000275 | 0 | 0 | 0 | 1 | 0 |
| AO090103000283 | 0 | 0 | 0 | 1 | 0 |
| AO090103000284 | 2 | 0 | 1 | 0 | 0 |
| AO090103000296 | 1 | 1 | 0 | 0 | 0 |
| AO090103000310 | 0 | 1 | 0 | 0 | 0 |
| AO090103000312 | 0 | 0 | 0 | 2 | 1 |
| AO090103000316 | 0 | 0 | 0 | 1 | 0 |
| AO090103000338 | 0 | 0 | 0 | 1 | 0 |
| AO090103000352 | 0 | 0 | 0 | 1 | 0 |
| AO090103000355 | 0 | 0 | 0 | 1 | 0 |
| AO090103000366 | 1 | 0 | 0 | 0 | 0 |
| AO090103000376 | 0 | 0 | 0 | 1 | 0 |
| AO090103000380 | 1 | 0 | 0 | 0 | 0 |
| AO090103000470 | 1 | 0 | 0 | 0 | 0 |
| AO090113000006 | 0 | 0 | 0 | 1 | 0 |
| AO090113000019 | 0 | 0 | 1 | 0 | 0 |
| AO090113000039 | 1 | 0 | 0 | 1 | 0 |
| AO090113000042 | 0 | 1 | 0 | 1 | 0 |
| AO090113000046 | 0 | 0 | 0 | 1 | 0 |
| AO090113000061 | 0 | 0 | 0 | 1 | 0 |
| AO090113000065 | 0 | 1 | 0 | 0 | 0 |
| AO090113000081 | 0 | 0 | 0 | 1 | 0 |
| AO090113000082 | 0 | 0 | 0 | 1 | 0 |
| AO090113000091 | 0 | 1 | 0 | 1 | 0 |
| AO090113000095 | 1 | 0 | 0 | 0 | 0 |
| AO090113000102 | 1 | 0 | 0 | 0 | 0 |
| AO090113000109 | 0 | 0 | 0 | 1 | 0 |
| AO090113000112 | 0 | 0 | 0 | 1 | 0 |
| AO090113000134 | 0 | 0 | 0 | 1 | 0 |
| AO090113000171 | 0 | 0 | 0 | 1 | 0 |
| AO090113000191 | 0 | 0 | 0 | 1 | 0 |
| AO090113000195 | 0 | 0 | 0 | 1 | 0 |
| AO090113000204 | 0 | 0 | 0 | 0 | 1 |
| AO090120000021 | 0 | 0 | 0 | 1 | 0 |
| AO090120000044 | 0 | 0 | 0 | 1 | 0 |
| AO090120000286 | 1 | 0 | 0 | 0 | 0 |
| AO090120000459 | 0 | 0 | 0 | 1 | 0 |
| AO090124000028 | 1 | 0 | 0 | 0 | 0 |
| AO090124000037 | 1 | 0 | 0 | 0 | 0 |
| AO090124000048 | 1 | 0 | 0 | 0 | 0 |
| AO090124000055 | 0 | 0 | 0 | 1 | 0 |
| AO090124000086 | 0 | 0 | 0 | 0 | 1 |
| AO090138000006 | 1 | 0 | 0 | 1 | 0 |
| AO090138000026 | 0 | 0 | 0 | 1 | 0 |
| AO090138000081 | 0 | 0 | 0 | 1 | 0 |
| AO090138000087 | 0 | 0 | 0 | 0 | 1 |
| AO090138000096 | 0 | 0 | 0 | 2 | 0 |
| AO090138000103 | 0 | 0 | 0 | 0 | 1 |
| AO090138000116 | 0 | 0 | 0 | 1 | 0 |
| AO090138000117 | 0 | 0 | 0 | 1 | 0 |
| AO090138000120 | 0 | 0 | 0 | 1 | 0 |
| AO090138000152 | 0 | 0 | 1 | 0 | 1 |
| AO090138000166 | 0 | 0 | 0 | 2 | 0 |
| AO090138000169 | 0 | 0 | 0 | 1 | 0 |
| AO090138000173 | 0 | 1 | 0 | 0 | 0 |
| AO090166000047 | 0 | 0 | 1 | 0 | 0 |
| AO090166000056 | 0 | 0 | 1 | 1 | 1 |
| AO090166000067 | 0 | 0 | 0 | 1 | 1 |
| AO090166000090 | 0 | 0 | 0 | 1 | 0 |
| AO090166000101 | 0 | 0 | 0 | 1 | 0 |
| AO090206000087 | 0 | 0 | 0 | 2 | 1 |
| AO090206000093 | 0 | 0 | 0 | 1 | 0 |
| AO090206000095 | 0 | 0 | 1 | 0 | 0 |
| AO090206000098 | 0 | 0 | 0 | 1 | 0 |
| AO090701000180 | 0 | 0 | 0 | 1 | 0 |
| AO090701000196 | 0 | 0 | 0 | 0 | 1 |
| AO090701000229 | 0 | 0 | 0 | 1 | 0 |
| AO090701000262 | 0 | 0 | 0 | 1 | 0 |
| AO090701000276 | 2 | 0 | 0 | 0 | 0 |
| AO090701000293 | 0 | 0 | 0 | 1 | 0 |
| AO090701000314 | 0 | 0 | 0 | 1 | 0 |
| AO090701000341 | 0 | 0 | 0 | 1 | 0 |
| AO090701000346 | 0 | 0 | 1 | 0 | 0 |
| AO090701000392 | 0 | 0 | 0 | 1 | 0 |
| AO090701000426 | 0 | 0 | 0 | 1 | 0 |
| AO090701000433 | 0 | 0 | 0 | 0 | 1 |
| AO090701000456 | 0 | 1 | 0 | 1 | 0 |
| AO090701000458 | 0 | 0 | 0 | 1 | 1 |
| AO090701000474 | 0 | 0 | 0 | 0 | 1 |
| AO090701000535 | 0 | 0 | 0 | 1 | 0 |
| AO090701000550 | 0 | 0 | 0 | 1 | 0 |
| AO090701000564 | 0 | 0 | 0 | 2 | 0 |
| AO090701000568 | 0 | 0 | 0 | 2 | 0 |
| AO090701000579 | 0 | 0 | 0 | 1 | 0 |
| AO090701000581 | 0 | 0 | 0 | 0 | 1 |
| AO090701000595 | 0 | 0 | 0 | 0 | 1 |
| AO090701000605 | 1 | 0 | 0 | 0 | 0 |
| AO090701000621 | 0 | 0 | 0 | 1 | 0 |
| AO090701000770 | 0 | 1 | 0 | 0 | 0 |
| AO090701000831 | 0 | 0 | 0 | 1 | 0 |
| AO090701000843 | 0 | 0 | 0 | 1 | 0 |
| AO090701000855 | 1 | 0 | 0 | 0 | 0 |
| AO090701000872 | 0 | 0 | 0 | 1 | 0 |
| AO090701000880 | 0 | 0 | 1 | 0 | 0 |
