# Supplementary material for: Comparison of Two Aspergillus oryzae Genomes From Different Clades Reveals Independent Evolution of Alpha-Amylase Duplication, Variation in Secondary Metabolism Genes, and Differences in Primary Metabolism
Source: Front Microbiol. 2021 Jul 13;12:691296. doi: 10.3389/fmicb.2021.691296 (PMC8313989; doi:10.3389/fmicb.2021.691296)
Supplement: Supplementary file 1 [file Data_Sheet_1.zip › Table 3.DOCX]

**Supplementary Table S3. Lineage specific genes in the *A. oryzae* RIB 40 genome in comparison to the 14160 genome.** Clusters represent neighboring lineage specific genes.

| **Cluster** | **Gene Number in Cluster** | **Gene ID** | **Chr** | **Start** | **Stop** |
| --- | --- | --- | --- | --- | --- |
| 1 | 1 | AO090009000002 | 1 | 9683 | 14221 |
| 1 | 2 | AO090009000003 | 1 | 15692 | 18301 |
| 1 | 3 | AO090009000006 | 1 | 23306 | 27898 |
| 1 | 4 | AO090009000008 | 1 | 33015 | 34673 |
| 1 | 5 | AO090009000009 | 1 | 38244 | 39400 |
| 1 | 6 | AO090009000010 | 1 | 40151 | 41365 |
| 1 | 7 | AO090009000011 | 1 | 41616 | 44348 |
| 1 | 8 | AO090009000012 | 1 | 45290 | 46639 |
| 1 | 9 | AO090009000014 | 1 | 50560 | 51939 |
| 1 | 10 | AO090009000015 | 1 | 53559 | 54571 |
| 1 | 11 | AO090009000016 | 1 | 54642 | 55184 |
| 1 | 12 | AO090009000017 | 1 | 57472 | 57942 |
| 1 | 13 | AO090009000018 | 1 | 62128 | 64279 |
| 1 | 14 | AO090009000019 | 1 | 64850 | 65937 |
| 2 | 1 | AO090009000179 | 1 | 480965 | 481582 |
| 2 | 2 | AO090009000180 | 1 | 481732 | 482224 |
| 2 | 3 | AO090009000181 | 1 | 482605 | 485014 |
| 2 | 4 | AO090009000182 | 1 | 485491 | 488048 |
| 3 | 1 | AO090009000515 | 1 | 1369504 | 1370246 |
| 3 | 2 | AO090009000516 | 1 | 1370880 | 1372706 |
| 3 | 3 | AO090009000517 | 1 | 1373936 | 1377215 |
| 3 | 4 | AO090009000519 | 1 | 1382096 | 1382874 |
| 4 | 1 | AO090009000569 | 1 | 1514859 | 1515473 |
| 4 | 2 | AO090009000570 | 1 | 1521997 | 1522506 |
| 5 | 1 | AO090005001598 | 1 | 2143768 | 2147905 |
| 5 | 2 | AO090005001597 | 1 | 2150728 | 2153716 |
| 6 | 1 | AO090005001359 | 1 | 2798875 | 2801919 |
| 7 | 1 | AO090005000959 | 1 | 3867011 | 3870168 |
| 7 | 2 | AO090005000958 | 1 | 3870842 | 3872322 |
| 7 | 3 | AO090005000957 | 1 | 3873485 | 3874787 |
| 7 | 4 | AO090005000956 | 1 | 3875108 | 3879030 |
| 7 | 5 | AO090005000955 | 1 | 3879734 | 3881444 |
| 7 | 6 | AO090005000954 | 1 | 3881664 | 3882724 |
| 7 | 7 | AO090005000953 | 1 | 3883226 | 3884770 |
| 7 | 8 | AO090005000952 | 1 | 3885365 | 3888400 |
| 7 | 9 | AO090005000951 | 1 | 3888778 | 3889383 |
| 8 | 1 | AO090005000675 | 1 | 4643701 | 4647061 |
| 8 | 2 | AO090005000674 | 1 | 4647139 | 4648140 |
| 9 | 1 | AO090005000348 | 1 | 5546704 | 5547556 |
| 10 | 1 | AO090005000214 | 1 | 5870474 | 5871352 |
| 10 | 2 | AO090005000213 | 1 | 5872279 | 5873015 |
| 10 | 3 | AO090005000211 | 1 | 5876309 | 5876781 |
| 10 | 4 | AO090005000210 | 1 | 5877428 | 5878430 |
| 11 | 1 | AO090005000042 | 1 | 6324101 | 6324892 |
| 11 | 2 | AO090005000041 | 1 | 6326386 | 6331395 |
| 12 | 1 | AO090308000002 | 1 | 6464231 | 6465804 |
| 12 | 2 | AO090308000003 | 1 | 6466631 | 6467884 |
| 12 | 3 | AO090308000004 | 1 | 6468314 | 6470139 |
| 12 | 4 | AO090308000007 | 1 | 6476637 | 6477050 |
| 12 | 5 | AO090308000008 | 1 | 6477752 | 6478319 |
| 12 | 6 | AO090308000009 | 1 | 6480275 | 6482625 |
| 12 | 7 | AO090308000010 | 1 | 6483170 | 6483701 |
| 12 | 8 | AO090308000011 | 1 | 6484615 | 6485209 |
| 13 | 1 | AO090308000017 | 1 | 6496094 | 6497232 |
| 13 | 2 | AO090308000018 | 1 | 6498878 | 6500424 |
| 13 | 3 | AO090308000019 | 1 | 6501021 | 6502591 |
| 13 | 4 | AO090308000020 | 1 | 6503373 | 6504353 |
| 13 | 5 | AO090308000021 | 1 | 6504868 | 6506955 |
| 13 | 6 | AO090308000022 | 1 | 6508708 | 6510551 |
| 14 | 1 | AO090001000255 | 2 | 623341 | 624168 |
| 14 | 2 | AO090001000256 | 2 | 626435 | 627372 |
| 14 | 3 | AO090001000257 | 2 | 628006 | 628813 |
| 14 | 4 | AO090001000258 | 2 | 629120 | 630472 |
| 14 | 5 | AO090001000259 | 2 | 631077 | 633254 |
| 15 | 1 | AO090001000528 | 2 | 1380093 | 1384033 |
| 15 | 2 | AO090001000529 | 2 | 1384423 | 1385965 |
| 16 | 1 | AO090001000534 | 2 | 1394201 | 1397498 |
| 16 | 2 | AO090001000535 | 2 | 1398731 | 1399671 |
| 16 | 3 | AO090001000536 | 2 | 1399725 | 1401134 |
| 16 | 4 | AO090001000537 | 2 | 1402374 | 1403087 |
| 16 | 5 | AO090001000538 | 2 | 1403178 | 1403958 |
| 16 | 6 | AO090001000539 | 2 | 1407715 | 1408257 |
| 16 | 7 | AO090001000540 | 2 | 1409079 | 1409923 |
| 17 | 1 | AO090003001310 | 2 | 5644334 | 5646066 |
| 17 | 2 | AO090003001312 | 2 | 5648076 | 5648598 |
| 17 | 3 | AO090003001313 | 2 | 5649029 | 5649857 |
| 18 | 1 | AO090003001434 | 2 | 5953987 | 5956340 |
| 18 | 2 | AO090003001435 | 2 | 5956467 | 5957189 |
| 18 | 3 | AO090003001436 | 2 | 5957855 | 5959516 |
| 18 | 4 | AO090003001437 | 2 | 5960074 | 5960698 |
| 18 | 5 | AO090003001438 | 2 | 5960781 | 5961560 |
| 18 | 6 | AO090003001440 | 2 | 5963355 | 5965290 |
| 18 | 7 | AO090003001441 | 2 | 5965869 | 5968140 |
| 18 | 8 | AO090003001442 | 2 | 5969128 | 5970057 |
| 18 | 9 | AO090003001443 | 2 | 5970780 | 5972312 |
| 18 | 10 | AO090003001444 | 2 | 5972839 | 5973648 |
| 18 | 11 | AO090003001445 | 2 | 5974286 | 5975154 |
| 18 | 12 | AO090003001446 | 2 | 5976640 | 5977382 |
| 18 | 13 | AO090003001448 | 2 | 5979316 | 5980465 |
| 18 | 14 | AO090003001449 | 2 | 5982760 | 5984046 |
| 18 | 15 | AO090003001450 | 2 | 5984454 | 5984888 |
| 18 | 16 | AO090003001451 | 2 | 5985108 | 5987122 |
| 18 | 17 | AO090003001452 | 2 | 5990288 | 5991269 |
| 18 | 18 | AO090003001453 | 2 | 5993003 | 5993965 |
| 18 | 19 | AO090003001455 | 2 | 5997083 | 5997881 |
| 19 | 1 | AO090003001469 | 2 | 6018192 | 6018957 |
| 20 | 1 | AO090003001556 | 2 | 6254240 | 6256001 |
| 20 | 2 | AO090003001558 | 2 | 6261999 | 6262503 |
| 20 | 3 | AO090023000001 | 3 | 1943 | 3463 |
| 20 | 4 | AO090023000002 | 3 | 4189 | 4966 |
| 20 | 5 | AO090023000003 | 3 | 6228 | 6920 |
| 20 | 6 | AO090023000004 | 3 | 7651 | 8039 |
| 20 | 7 | AO090023000006 | 3 | 11234 | 13940 |
| 20 | 8 | AO090023000007 | 3 | 16001 | 19716 |
| 20 | 9 | AO090023000008 | 3 | 21131 | 22662 |
| 20 | 10 | AO090023000010 | 3 | 26595 | 27755 |
| 20 | 11 | AO090023000011 | 3 | 29813 | 31114 |
| 20 | 12 | AO090023000012 | 3 | 32362 | 35300 |
| 20 | 13 | AO090023000013 | 3 | 37175 | 38521 |
| 20 | 14 | AO090023000014 | 3 | 38934 | 39578 |
| 20 | 15 | AO090023000015 | 3 | 41238 | 42632 |
| 20 | 16 | AO090023000016 | 3 | 43863 | 46717 |
| 20 | 17 | AO090023000017 | 3 | 47536 | 48006 |
| 20 | 18 | AO090023000018 | 3 | 48481 | 49868 |
| 20 | 19 | AO090023000019 | 3 | 50819 | 52601 |
| 20 | 20 | AO090023000021 | 3 | 55159 | 56100 |
| 20 | 21 | AO090023000022 | 3 | 59624 | 60702 |
| 20 | 22 | AO090023000023 | 3 | 62698 | 64568 |
| 20 | 23 | AO090023000024 | 3 | 65612 | 65999 |
| 20 | 24 | AO090023000025 | 3 | 68686 | 70662 |
| 20 | 25 | AO090023000026 | 3 | 71757 | 73440 |
| 20 | 26 | AO090023000027 | 3 | 76026 | 77193 |
| 20 | 27 | AO090023000028 | 3 | 78438 | 79286 |
| 20 | 28 | AO090023000029 | 3 | 81682 | 82369 |
| 20 | 29 | AO090023000031 | 3 | 84797 | 87160 |
| 20 | 30 | AO090023000032 | 3 | 87403 | 87874 |
| 20 | 31 | AO090023000033 | 3 | 88577 | 89245 |
| 20 | 32 | AO090023000034 | 3 | 89925 | 90947 |
| 20 | 33 | AO090023000035 | 3 | 92047 | 93000 |
| 20 | 34 | AO090023000036 | 3 | 93970 | 95248 |
| 21 | 1 | AO090023000183 | 3 | 454934 | 455479 |
| 21 | 2 | AO090023000184 | 3 | 457519 | 458004 |
| 22 | 1 | AO090023000188 | 3 | 469566 | 471131 |
| 22 | 2 | AO090023000189 | 3 | 472501 | 475264 |
| 22 | 3 | AO090023000190 | 3 | 475673 | 476381 |
| 22 | 4 | AO090023000191 | 3 | 477849 | 479612 |
| 22 | 5 | AO090023000192 | 3 | 480458 | 482071 |
| 23 | 1 | AO090023000248 | 3 | 632261 | 632875 |
| 23 | 2 | AO090023000249 | 3 | 640337 | 641431 |
| 23 | 3 | AO090023000250 | 3 | 641713 | 643806 |
| 24 | 1 | AO090023000410 | 3 | 1049669 | 1051512 |
| 24 | 2 | AO090023000411 | 3 | 1053862 | 1055321 |
| 25 | 1 | AO090023000456 | 3 | 1170838 | 1172488 |
| 25 | 2 | AO090023000457 | 3 | 1172850 | 1173800 |
| 25 | 3 | AO090023000458 | 3 | 1174739 | 1177580 |
| 26 | 1 | AO090023000824 | 3 | 2148738 | 2149607 |
| 26 | 2 | AO090023000825 | 3 | 2151861 | 2153788 |
| 27 | 1 | AO090023001009 | 3 | 2654075 | 2654654 |
| 28 | 1 | AO090026000586 | 3 | 3487212 | 3489426 |
| 28 | 2 | AO090026000585 | 3 | 3489600 | 3493616 |
| 28 | 3 | AO090026000584 | 3 | 3494588 | 3496126 |
| 28 | 4 | AO090026000582 | 3 | 3497500 | 3498575 |
| 28 | 5 | AO090026000581 | 3 | 3499537 | 3501251 |
| 28 | 6 | AO090026000580 | 3 | 3502315 | 3503100 |
| 28 | 7 | AO090026000579 | 3 | 3503521 | 3505086 |
| 28 | 8 | AO090026000578 | 3 | 3505675 | 3507180 |
| 28 | 9 | AO090026000577 | 3 | 3507793 | 3509610 |
| 28 | 10 | AO090026000576 | 3 | 3510111 | 3510716 |
| 28 | 11 | AO090026000575 | 3 | 3510777 | 3513098 |
| 29 | 1 | AO090026000566 | 3 | 3536042 | 3538156 |
| 29 | 2 | AO090026000565 | 3 | 3538296 | 3539978 |
| 30 | 1 | AO090026000178 | 3 | 4618497 | 4619072 |
| 30 | 2 | AO090026000177 | 3 | 4620212 | 4621336 |
| 30 | 3 | AO090026000175 | 3 | 4624284 | 4624663 |
| 30 | 4 | AO090026000174 | 3 | 4630813 | 4631398 |
| 30 | 5 | AO090026000173 | 3 | 4632633 | 4634063 |
| 30 | 6 | AO090026000169 | 3 | 4640158 | 4641115 |
| 31 | 1 | AO090026000137 | 3 | 4732670 | 4733354 |
| 31 | 2 | AO090026000136 | 3 | 4735184 | 4739033 |
| 32 | 1 | AO090026000027 | 3 | 5048567 | 5050517 |
| 32 | 2 | AO090026000026 | 3 | 5051763 | 5053249 |
| 32 | 3 | AO090026000025 | 3 | 5054515 | 5055849 |
| 32 | 4 | AO090026000024 | 3 | 5056027 | 5056875 |
| 32 | 5 | AO090026000023 | 3 | 5057080 | 5057537 |
| 32 | 6 | AO090026000022 | 3 | 5057912 | 5059468 |
| 32 | 7 | AO090026000021 | 3 | 5059836 | 5061427 |
| 32 | 8 | AO090026000020 | 3 | 5062479 | 5064014 |
| 32 | 9 | AO090026000019 | 3 | 5065046 | 5065945 |
| 32 | 10 | AO090026000018 | 3 | 5067039 | 5068043 |
| 32 | 11 | AO090026000017 | 3 | 5068334 | 5069849 |
| 32 | 12 | AO090026000016 | 3 | 5070256 | 5071092 |
| 32 | 13 | AO090026000015 | 3 | 5071654 | 5073104 |
| 32 | 14 | AO090026000014 | 3 | 5073844 | 5075178 |
| 32 | 15 | AO090026000013 | 3 | 5076457 | 5082303 |
| 32 | 16 | AO090026000012 | 3 | 5082993 | 5088137 |
| 32 | 17 | AO090026000011 | 3 | 5089452 | 5090441 |
| 32 | 18 | AO090026000010 | 3 | 5090746 | 5091377 |
| 32 | 19 | AO090026000009 | 3 | 5092130 | 5098753 |
| 32 | 20 | AO090026000008 | 3 | 5100094 | 5101987 |
| 32 | 21 | AO090026000006 | 3 | 5105308 | 5105975 |
| 32 | 22 | AO090026000005 | 3 | 5108316 | 5111016 |
| 32 | 23 | AO090026000004 | 3 | 5112805 | 5114033 |
| 32 | 24 | AO090026000003 | 3 | 5115280 | 5116647 |
| 32 | 25 | AO090026000002 | 3 | 5117116 | 5118493 |
| 32 | 26 | AO090026000001 | 3 | 5121104 | 5122857 |
| 33 | 1 | AO090012000002 | 4 | 9409 | 11379 |
| 34 | 1 | AO090012000055 | 4 | 128969 | 129583 |
| 34 | 2 | AO090012000056 | 4 | 129841 | 131480 |
| 34 | 3 | AO090012000058 | 4 | 132333 | 134905 |
| 35 | 1 | AO090012000116 | 4 | 286592 | 287471 |
| 35 | 2 | AO090012000117 | 4 | 289672 | 290517 |
| 35 | 3 | AO090012000119 | 4 | 292257 | 293167 |
| 36 | 1 | AO090012000411 | 4 | 1007647 | 1008137 |
| 37 | 1 | AO090012000768 | 4 | 1967508 | 1969089 |
| 38 | 1 | AO090102000538 | 4 | 3064766 | 3066544 |
| 39 | 1 | AO090102000466 | 4 | 3260874 | 3263913 |
| 39 | 2 | AO090102000465 | 4 | 3264838 | 3273873 |
| 39 | 3 | AO090102000464 | 4 | 3275088 | 3276134 |
| 39 | 4 | AO090102000461 | 4 | 3278697 | 3280492 |
| 39 | 5 | AO090102000460 | 4 | 3283568 | 3284970 |
| 40 | 1 | AO090102000441 | 4 | 3338330 | 3341421 |
| 40 | 2 | AO090102000440 | 4 | 3343326 | 3343807 |
| 40 | 3 | AO090102000439 | 4 | 3345158 | 3345632 |
| 40 | 4 | AO090102000438 | 4 | 3349600 | 3351515 |
| 40 | 5 | AO090102000436 | 4 | 3353235 | 3353618 |
| 40 | 6 | AO090102000435 | 4 | 3354080 | 3355030 |
| 40 | 7 | AO090102000434 | 4 | 3355200 | 3355994 |
| 40 | 8 | AO090102000433 | 4 | 3356808 | 3358761 |
| 40 | 9 | AO090102000431 | 4 | 3359491 | 3360321 |
| 40 | 10 | AO090102000429 | 4 | 3361429 | 3363207 |
| 40 | 11 | AO090102000428 | 4 | 3363880 | 3364584 |
| 41 | 1 | AO090102000421 | 4 | 3379675 | 3382963 |
| 41 | 2 | AO090102000419 | 4 | 3386437 | 3386933 |
| 41 | 3 | AO090102000418 | 4 | 3388157 | 3390622 |
| 42 | 1 | AO090102000319 | 4 | 3677485 | 3678114 |
| 42 | 2 | AO090102000318 | 4 | 3679389 | 3681018 |
| 43 | 1 | AO090102000285 | 4 | 3750705 | 3751460 |
| 43 | 2 | AO090102000284 | 4 | 3753041 | 3753836 |
| 43 | 3 | AO090102000283 | 4 | 3754640 | 3755657 |
| 44 | 1 | AO090166000002 | 4 | 4553209 | 4553850 |
| 44 | 2 | AO090166000003 | 4 | 4555472 | 4556758 |
| 44 | 3 | AO090166000004 | 4 | 4560030 | 4562819 |
| 44 | 4 | AO090166000005 | 4 | 4563732 | 4564532 |
| 44 | 5 | AO090166000006 | 4 | 4566158 | 4567285 |
| 44 | 6 | AO090166000008 | 4 | 4570426 | 4571943 |
| 44 | 7 | AO090166000009 | 4 | 4575441 | 4576666 |
| 44 | 8 | AO090166000010 | 4 | 4579773 | 4581045 |
| 44 | 9 | AO090166000012 | 4 | 4586428 | 4587024 |
| 44 | 10 | AO090166000013 | 4 | 4587097 | 4587777 |
| 44 | 11 | AO090166000015 | 4 | 4596676 | 4599761 |
| 44 | 12 | AO090166000016 | 4 | 4602198 | 4602914 |
| 44 | 13 | AO090166000017 | 4 | 4603574 | 4605652 |
| 44 | 14 | AO090166000019 | 4 | 4608508 | 4608890 |
| 44 | 15 | AO090166000020 | 4 | 4614026 | 4615942 |
| 44 | 16 | AO090166000022 | 4 | 4619591 | 4620741 |
| 44 | 17 | AO090166000023 | 4 | 4622828 | 4623246 |
| 44 | 18 | AO090166000024 | 4 | 4623488 | 4624186 |
| 44 | 19 | AO090166000025 | 4 | 4625519 | 4626189 |
| 44 | 20 | AO090166000026 | 4 | 4626960 | 4628985 |
| 44 | 21 | AO090166000027 | 4 | 4632693 | 4633098 |
| 44 | 22 | AO090166000029 | 4 | 4635703 | 4638564 |
| 44 | 23 | AO090166000030 | 4 | 4641095 | 4642050 |
| 44 | 24 | AO090166000031 | 4 | 4642878 | 4643273 |
| 44 | 25 | AO090166000032 | 4 | 4644286 | 4645095 |
| 44 | 26 | AO090166000034 | 4 | 4647395 | 4648097 |
| 44 | 27 | AO090166000035 | 4 | 4648687 | 4649235 |
| 44 | 28 | AO090166000036 | 4 | 4649453 | 4650643 |
| 45 | 1 | AO090166000039 | 4 | 4654041 | 4654460 |
| 45 | 2 | AO090166000040 | 4 | 4655109 | 4656289 |
| 45 | 3 | AO090166000042 | 4 | 4659225 | 4660158 |
| 45 | 4 | AO090166000043 | 4 | 4661197 | 4663733 |
| 45 | 5 | AO090166000044 | 4 | 4665290 | 4667127 |
| 45 | 6 | AO090166000045 | 4 | 4667444 | 4668411 |
| 46 | 1 | AO090166000058 | 4 | 4709941 | 4711709 |
| 47 | 1 | AO090166000116 | 4 | 4859487 | 4861069 |
| 47 | 2 | AO090166000117 | 4 | 4864571 | 4866494 |
| 47 | 3 | AO090166000118 | 4 | 4867715 | 4868398 |
| 47 | 4 | AO090166000119 | 4 | 4868667 | 4870474 |
| 47 | 5 | AO090166000120 | 4 | 4871343 | 4872188 |
| 47 | 6 | AO090166000121 | 4 | 4873020 | 4873866 |
| 47 | 7 | AO090166000122 | 4 | 4874352 | 4875509 |
| 47 | 8 | AO090166000123 | 4 | 4877903 | 4883545 |
| 47 | 9 | AO090701000907 | 5 | 21 | 611 |
| 47 | 10 | AO090701000906 | 5 | 1004 | 3765 |
| 47 | 11 | AO090701000905 | 5 | 4418 | 6182 |
| 47 | 12 | AO090701000904 | 5 | 6749 | 7600 |
| 47 | 13 | AO090701000902 | 5 | 11206 | 12577 |
| 47 | 14 | AO090701000900 | 5 | 13697 | 16560 |
| 47 | 15 | AO090701000899 | 5 | 18273 | 21398 |
| 48 | 1 | AO090701000857 | 5 | 113118 | 113440 |
| 49 | 1 | AO090701000698 | 5 | 527442 | 528968 |
| 49 | 2 | AO090701000697 | 5 | 529977 | 531569 |
| 50 | 1 | AO090701000575 | 5 | 829296 | 830093 |
| 51 | 1 | AO090701000568 | 5 | 853868 | 855281 |
| 51 | 2 | AO090701000567 | 5 | 855662 | 858567 |
| 51 | 3 | AO090701000566 | 5 | 858577 | 859901 |
| 52 | 1 | AO090701000262 | 5 | 1654394 | 1655866 |
| 52 | 2 | AO090701000260 | 5 | 1656736 | 1657501 |
| 52 | 3 | AO090701000259 | 5 | 1658393 | 1659639 |
| 52 | 4 | AO090701000257 | 5 | 1661809 | 1662523 |
| 52 | 5 | AO090701000255 | 5 | 1664154 | 1664569 |
| 53 | 1 | AO090124000037 | 5 | 2592899 | 2594239 |
| 53 | 2 | AO090124000036 | 5 | 2594871 | 2597373 |
| 53 | 3 | AO090124000035 | 5 | 2598686 | 2601468 |
| 53 | 4 | AO090124000033 | 5 | 2603157 | 2603834 |
| 53 | 5 | AO090124000032 | 5 | 2604797 | 2605483 |
| 53 | 6 | AO090124000031 | 5 | 2606150 | 2607529 |
| 54 | 1 | AO090113000019 | 5 | 4093715 | 4097070 |
| 54 | 2 | AO090113000020 | 5 | 4097241 | 4098302 |
| 54 | 3 | AO090113000021 | 5 | 4100195 | 4103872 |
| 55 | 1 | AO090113000057 | 5 | 4181814 | 4184138 |
| 55 | 2 | AO090113000058 | 5 | 4184694 | 4187851 |
| 55 | 3 | AO090113000059 | 5 | 4188603 | 4190582 |
| 56 | 1 | AO090113000091 | 5 | 4270414 | 4273823 |
| 56 | 2 | AO090113000092 | 5 | 4274498 | 4275535 |
| 56 | 3 | AO090113000095 | 5 | 4280503 | 4281172 |
| 57 | 1 | AO090020000374 | 6 | 898655 | 900197 |
| 57 | 2 | AO090020000373 | 6 | 902365 | 904096 |
| 57 | 3 | AO090020000372 | 6 | 904144 | 905607 |
| 57 | 4 | AO090020000371 | 6 | 906174 | 906839 |
| 57 | 5 | AO090020000370 | 6 | 907353 | 907967 |
| 57 | 6 | AO090020000369 | 6 | 908870 | 912397 |
| 57 | 7 | AO090020000368 | 6 | 913834 | 914364 |
| 57 | 8 | AO090020000367 | 6 | 914979 | 916370 |
| 57 | 9 | AO090020000366 | 6 | 916686 | 917990 |
| 57 | 10 | AO090020000365 | 6 | 918808 | 922008 |
| 57 | 11 | AO090020000364 | 6 | 922783 | 924528 |
| 57 | 12 | AO090020000363 | 6 | 925365 | 926550 |
| 57 | 13 | AO090020000362 | 6 | 927272 | 928975 |
| 57 | 14 | AO090020000361 | 6 | 929577 | 932837 |
| 57 | 15 | AO090020000360 | 6 | 933986 | 938482 |
| 57 | 16 | AO090020000357 | 6 | 939879 | 941490 |
| 58 | 1 | AO090020000310 | 6 | 1057250 | 1059266 |
| 58 | 2 | AO090020000309 | 6 | 1059999 | 1060692 |
| 58 | 3 | AO090020000308 | 6 | 1061615 | 1062052 |
| 59 | 1 | AO090020000261 | 6 | 1166960 | 1168770 |
| 60 | 1 | AO090020000091 | 6 | 1574228 | 1578187 |
| 60 | 2 | AO090020000089 | 6 | 1579999 | 1581166 |
| 61 | 1 | AO090038000511 | 6 | 2292846 | 2297241 |
| 61 | 2 | AO090038000509 | 6 | 2300988 | 2303373 |
| 61 | 3 | AO090038000508 | 6 | 2303753 | 2306046 |
| 61 | 4 | AO090038000507 | 6 | 2306323 | 2307487 |
| 62 | 1 | AO090038000151 | 6 | 3290179 | 3291327 |
| 62 | 2 | AO090038000150 | 6 | 3291406 | 3293667 |
| 62 | 3 | AO090038000149 | 6 | 3294048 | 3296935 |
| 62 | 4 | AO090038000148 | 6 | 3298354 | 3300075 |
| 62 | 5 | AO090038000147 | 6 | 3300529 | 3302130 |
| 62 | 6 | AO090038000146 | 6 | 3302997 | 3303768 |
| 63 | 1 | AO090038000107 | 6 | 3394932 | 3397217 |
| 63 | 2 | AO090038000106 | 6 | 3397891 | 3398548 |
| 63 | 3 | AO090038000105 | 6 | 3401072 | 3401863 |
| 64 | 1 | AO090038000007 | 6 | 3639534 | 3640013 |
| 64 | 2 | AO090038000004 | 6 | 3651581 | 3652048 |
| 64 | 3 | AO090038000003 | 6 | 3652137 | 3652760 |
| 64 | 4 | AO090038000002 | 6 | 3653062 | 3653830 |
| 65 | 1 | AO090138000202 | 6 | 3698691 | 3703192 |
| 65 | 2 | AO090138000201 | 6 | 3705627 | 3707696 |
| 65 | 3 | AO090138000200 | 6 | 3709075 | 3710652 |
| 65 | 4 | AO090138000199 | 6 | 3710772 | 3711673 |
| 65 | 5 | AO090138000198 | 6 | 3712793 | 3714592 |
| 65 | 6 | AO090138000196 | 6 | 3718594 | 3720342 |
| 66 | 1 | AO090138000182 | 6 | 3751822 | 3752248 |
| 66 | 2 | AO090138000181 | 6 | 3752603 | 3753127 |
| 66 | 3 | AO090138000180 | 6 | 3754293 | 3755335 |
| 66 | 4 | AO090138000179 | 6 | 3758302 | 3759771 |
| 67 | 1 | AO090138000161 | 6 | 3811947 | 3812701 |
| 67 | 2 | AO090138000160 | 6 | 3813652 | 3815799 |
| 67 | 3 | AO090138000159 | 6 | 3816005 | 3817182 |
| 67 | 4 | AO090138000157 | 6 | 3819956 | 3820823 |
| 68 | 1 | AO090011000001 | 7 | 3798 | 4716 |
| 68 | 2 | AO090011000002 | 7 | 4937 | 5848 |
| 68 | 3 | AO090011000004 | 7 | 6608 | 7506 |
| 68 | 4 | AO090011000005 | 7 | 8086 | 8922 |
| 68 | 5 | AO090011000006 | 7 | 9357 | 10105 |
| 68 | 6 | AO090011000007 | 7 | 10195 | 11005 |
| 68 | 7 | AO090011000008 | 7 | 11859 | 13263 |
| 68 | 8 | AO090011000009 | 7 | 15322 | 16771 |
| 68 | 9 | AO090011000010 | 7 | 18383 | 19625 |
| 68 | 10 | AO090011000011 | 7 | 21450 | 23832 |
| 68 | 11 | AO090011000012 | 7 | 25245 | 28910 |
| 68 | 12 | AO090011000013 | 7 | 29127 | 29522 |
| 68 | 13 | AO090011000014 | 7 | 31570 | 33318 |
| 68 | 14 | AO090011000015 | 7 | 33837 | 41492 |
| 68 | 15 | AO090011000016 | 7 | 42160 | 43011 |
| 68 | 16 | AO090011000017 | 7 | 43815 | 46139 |
| 68 | 17 | AO090011000019 | 7 | 48102 | 52051 |
| 68 | 18 | AO090011000020 | 7 | 53603 | 55930 |
| 68 | 19 | AO090011000021 | 7 | 56616 | 57457 |
| 68 | 20 | AO090011000022 | 7 | 60265 | 61362 |
| 68 | 21 | AO090011000023 | 7 | 61854 | 64643 |
| 69 | 1 | AO090011000031 | 7 | 82097 | 84347 |
| 70 | 1 | AO090011000092 | 7 | 262325 | 264092 |
| 71 | 1 | AO090011000310 | 7 | 786052 | 787938 |
| 71 | 2 | AO090011000311 | 7 | 789036 | 789605 |
| 71 | 3 | AO090011000313 | 7 | 792318 | 792745 |
| 72 | 1 | AO090011000687 | 7 | 1766536 | 1767654 |
| 72 | 2 | AO090011000688 | 7 | 1767872 | 1768663 |
| 72 | 3 | AO090011000689 | 7 | 1771946 | 1773401 |
| 73 | 1 | AO090011000863 | 7 | 2209058 | 2211505 |
| 73 | 2 | AO090011000864 | 7 | 2212463 | 2212915 |
| 73 | 3 | AO090011000865 | 7 | 2212967 | 2214910 |
| 73 | 4 | AO090011000866 | 7 | 2215600 | 2218285 |
| 74 | 1 | AO090206000109 | 7 | 2864363 | 2868700 |
| 75 | 1 | AO090103000509 | 8 | 8670 | 9284 |
| 75 | 2 | AO090103000508 | 8 | 14160 | 15265 |
| 75 | 3 | AO090103000507 | 8 | 15409 | 17004 |
| 75 | 4 | AO090103000506 | 8 | 18106 | 19897 |
| 75 | 5 | AO090103000504 | 8 | 21200 | 21867 |
| 75 | 6 | AO090103000503 | 8 | 21915 | 22580 |
| 75 | 7 | AO090103000502 | 8 | 22741 | 23317 |
| 75 | 8 | AO090103000499 | 8 | 27078 | 29710 |
| 76 | 1 | AO090103000310 | 8 | 479115 | 480279 |
| 76 | 2 | AO090103000309 | 8 | 481163 | 481925 |
| 76 | 3 | AO090103000308 | 8 | 481937 | 483187 |
| 76 | 4 | AO090103000307 | 8 | 483681 | 484489 |
| 76 | 5 | AO090103000306 | 8 | 485934 | 486643 |
| 76 | 6 | AO090103000304 | 8 | 488021 | 488397 |
| 76 | 7 | AO090103000303 | 8 | 488702 | 492082 |
| 76 | 8 | AO090103000302 | 8 | 494469 | 496175 |
| 77 | 1 | AO090103000219 | 8 | 712556 | 714145 |
| 77 | 2 | AO090103000218 | 8 | 714749 | 717914 |
| 77 | 3 | AO090103000217 | 8 | 721450 | 722511 |
| 77 | 4 | AO090103000216 | 8 | 723139 | 724923 |
| 77 | 5 | AO090103000215 | 8 | 725145 | 725794 |
| 78 | 1 | AO090103000212 | 8 | 743460 | 744432 |
| 79 | 1 | AO090103000146 | 8 | 889376 | 891134 |
| 79 | 2 | AO090103000145 | 8 | 891473 | 893246 |
| 79 | 3 | AO090103000144 | 8 | 893800 | 894507 |
| 79 | 4 | AO090103000142 | 8 | 896732 | 897976 |
| 80 | 1 | AO090103000001 | 8 | 1272543 | 1276776 |
| 81 | 1 | AO090010000737 | 8 | 1485677 | 1487065 |
| 81 | 2 | AO090010000736 | 8 | 1487448 | 1489124 |
| 81 | 3 | AO090010000735 | 8 | 1491369 | 1493041 |
| 81 | 4 | AO090010000734 | 8 | 1493304 | 1493925 |
| 81 | 5 | AO090010000733 | 8 | 1495083 | 1496636 |
| 81 | 6 | AO090010000732 | 8 | 1499000 | 1501136 |
| 82 | 1 | AO090010000696 | 8 | 1583654 | 1584981 |
| 82 | 2 | AO090010000695 | 8 | 1585435 | 1586002 |
| 82 | 3 | AO090010000694 | 8 | 1589796 | 1591337 |
| 82 | 4 | AO090010000693 | 8 | 1592457 | 1594053 |
| 82 | 5 | AO090010000692 | 8 | 1595274 | 1596301 |
| 82 | 6 | AO090010000691 | 8 | 1596791 | 1600872 |
| 83 | 1 | AO090010000571 | 8 | 1882722 | 1885559 |
| 83 | 2 | AO090010000570 | 8 | 1887797 | 1888936 |
| 83 | 3 | AO090010000569 | 8 | 1890547 | 1891841 |
| 84 | 1 | AO090010000315 | 8 | 2501808 | 2502755 |
| 85 | 1 | AO090010000302 | 8 | 2544861 | 2547372 |
| 85 | 2 | AO090010000301 | 8 | 2547508 | 2548541 |
| 86 | 1 | AO090010000299 | 8 | 2563646 | 2564295 |
| 86 | 2 | AO090010000298 | 8 | 2564597 | 2565043 |
| 86 | 3 | AO090010000297 | 8 | 2565451 | 2568042 |
| 86 | 4 | AO090010000296 | 8 | 2568815 | 2569828 |
| 86 | 5 | AO090010000294 | 8 | 2579121 | 2582513 |
| 86 | 6 | AO090010000293 | 8 | 2587684 | 2589502 |
| 86 | 7 | AO090010000292 | 8 | 2590958 | 2594725 |
| 87 | 1 | AO090010000002 | 8 | 3385254 | 3389638 |
| 87 | 2 | AO090010000001 | 8 | 3390335 | 3392149 |
