# Supplementary material for: Comparison of Two Aspergillus oryzae Genomes From Different Clades Reveals Independent Evolution of Alpha-Amylase Duplication, Variation in Secondary Metabolism Genes, and Differences in Primary Metabolism
Source: Front Microbiol. 2021 Jul 13;12:691296. doi: 10.3389/fmicb.2021.691296 (PMC8313989; doi:10.3389/fmicb.2021.691296)
Supplement: Supplementary file 1 [file Data_Sheet_1.zip › Table 4.DOCX]

**Supplementary Table S4. Lineage specific genes in the *A. oryzae* 14160 genome in comparison to the RIB 40 genome.** Clusters represent neighboring lineage specific genes.

| **Cluster** | **Gene Number in Cluster** | **Gene ID** | **Scaffold** | **Start** | **Stop** |
| --- | --- | --- | --- | --- | --- |
| 1 | 1 | FUN_000084 | contig_1 | 219763 | 221543 |
| 2 | 1 | FUN_000138 | contig_1 | 372727 | 373980 |
| 3 | 1 | FUN_000354 | contig_1 | 977435 | 981952 |
| 4 | 1 | FUN_000548 | contig_1 | 1602089 | 1602179 |
| 4 | 2 | FUN_000549 | contig_1 | 1610319 | 1610845 |
| 4 | 3 | FUN_000550 | contig_1 | 1612076 | 1614715 |
| 5 | 1 | FUN_000792 | contig_1 | 2291215 | 2292438 |
| 6 | 1 | FUN_005495 | contig_2 | 137905 | 140832 |
| 6 | 2 | FUN_005496 | contig_2 | 141376 | 145188 |
| 7 | 1 | FUN_005498 | contig_2 | 150447 | 154717 |
| 7 | 2 | FUN_005499 | contig_2 | 157504 | 158843 |
| 7 | 3 | FUN_005500 | contig_2 | 159681 | 160875 |
| 7 | 4 | FUN_005501 | contig_2 | 163286 | 164589 |
| 7 | 5 | FUN_005502 | contig_2 | 166056 | 167702 |
| 7 | 6 | FUN_005503 | contig_2 | 168062 | 170059 |
| 8 | 1 | FUN_005537 | contig_2 | 263517 | 265568 |
| 8 | 2 | FUN_005538 | contig_2 | 265933 | 267458 |
| 8 | 3 | FUN_005539 | contig_2 | 267864 | 269700 |
| 9 | 1 | FUN_005676 | contig_2 | 640921 | 642880 |
| 9 | 2 | FUN_005677 | contig_2 | 644579 | 645439 |
| 9 | 3 | FUN_005678 | contig_2 | 646149 | 651020 |
| 9 | 4 | FUN_005679 | contig_2 | 651220 | 652263 |
| 10 | 1 | FUN_005704 | contig_2 | 732702 | 733121 |
| 10 | 2 | FUN_005705 | contig_2 | 733671 | 734616 |
| 10 | 3 | FUN_005706 | contig_2 | 735476 | 735991 |
| 11 | 1 | FUN_005899 | contig_2 | 1381214 | 1382613 |
| 11 | 2 | FUN_005900 | contig_2 | 1386487 | 1386975 |
| 11 | 3 | FUN_005901 | contig_2 | 1389488 | 1390462 |
| 11 | 4 | FUN_005902 | contig_2 | 1393525 | 1401486 |
| 11 | 5 | FUN_005903 | contig_2 | 1402733 | 1403855 |
| 11 | 6 | FUN_005904 | contig_2 | 1405640 | 1406628 |
| 12 | 1 | FUN_005908 | contig_2 | 1416237 | 1419115 |
| 13 | 1 | FUN_006038 | contig_2 | 1805248 | 1806390 |
| 14 | 1 | FUN_007295 | contig_3 | 274362 | 275607 |
| 14 | 2 | FUN_007296 | contig_3 | 276667 | 277533 |
| 14 | 3 | FUN_007297 | contig_3 | 285072 | 285155 |
| 15 | 1 | FUN_007351 | contig_3 | 458269 | 459297 |
| 15 | 2 | FUN_007352 | contig_3 | 461200 | 462601 |
| 15 | 3 | FUN_007353 | contig_3 | 464853 | 465393 |
| 15 | 4 | FUN_007354 | contig_3 | 467599 | 469149 |
| 15 | 5 | FUN_007355 | contig_3 | 472674 | 474425 |
| 15 | 6 | FUN_007356 | contig_3 | 479843 | 480907 |
| 16 | 1 | FUN_007422 | contig_3 | 693436 | 695057 |
| 16 | 2 | FUN_007423 | contig_3 | 695532 | 696599 |
| 16 | 3 | FUN_007424 | contig_3 | 698768 | 699256 |
| 16 | 4 | FUN_007425 | contig_3 | 700208 | 700729 |
| 17 | 1 | FUN_007484 | contig_3 | 889744 | 891556 |
| 17 | 2 | FUN_007485 | contig_3 | 893382 | 894725 |
| 17 | 3 | FUN_007486 | contig_3 | 896013 | 897545 |
| 18 | 1 | FUN_007495 | contig_3 | 952138 | 953397 |
| 19 | 1 | FUN_007507 | contig_3 | 987790 | 988408 |
| 19 | 2 | FUN_007508 | contig_3 | 990142 | 991386 |
| 20 | 1 | FUN_007821 | contig_4 | 493964 | 495358 |
| 20 | 2 | FUN_007822 | contig_4 | 496963 | 498143 |
| 20 | 3 | FUN_007823 | contig_4 | 499332 | 500393 |
| 21 | 1 | FUN_007839 | contig_4 | 548262 | 549209 |
| 21 | 2 | FUN_007840 | contig_4 | 550519 | 551301 |
| 22 | 1 | FUN_007851 | contig_4 | 574825 | 576220 |
| 22 | 2 | FUN_007852 | contig_4 | 576713 | 578394 |
| 23 | 1 | FUN_007964 | contig_4 | 910777 | 918344 |
| 23 | 2 | FUN_007965 | contig_4 | 919915 | 920751 |
| 23 | 3 | FUN_007966 | contig_4 | 921058 | 921929 |
| 24 | 1 | FUN_007969 | contig_4 | 927749 | 929140 |
| 25 | 1 | FUN_007979 | contig_4 | 946326 | 947693 |
| 25 | 2 | FUN_007980 | contig_4 | 948532 | 950093 |
| 26 | 1 | FUN_008191 | contig_4 | 1571721 | 1572139 |
| 26 | 2 | FUN_008192 | contig_4 | 1572202 | 1573405 |
| 26 | 3 | FUN_008193 | contig_4 | 1574383 | 1574859 |
| 27 | 1 | FUN_008275 | contig_5 | 37626 | 38660 |
| 28 | 1 | FUN_008380 | contig_5 | 326334 | 327323 |
| 28 | 2 | FUN_008381 | contig_5 | 328761 | 329255 |
| 29 | 1 | FUN_008412 | contig_5 | 428578 | 429207 |
| 29 | 2 | FUN_008413 | contig_5 | 431935 | 432891 |
| 29 | 3 | FUN_008414 | contig_5 | 436909 | 437337 |
| 29 | 4 | FUN_008415 | contig_5 | 442972 | 445254 |
| 29 | 5 | FUN_008416 | contig_5 | 460576 | 462318 |
| 29 | 6 | FUN_008417 | contig_5 | 464479 | 465498 |
| 29 | 7 | FUN_008418 | contig_5 | 465804 | 466785 |
| 29 | 8 | FUN_008419 | contig_5 | 467083 | 471357 |
| 29 | 9 | FUN_008420 | contig_5 | 472619 | 474418 |
| 29 | 10 | FUN_008421 | contig_5 | 483782 | 484689 |
| 29 | 11 | FUN_008422 | contig_5 | 487283 | 489208 |
| 29 | 12 | FUN_008423 | contig_5 | 490127 | 491496 |
| 29 | 13 | FUN_008424 | contig_5 | 492387 | 492968 |
| 29 | 14 | FUN_008425 | contig_5 | 493618 | 494743 |
| 30 | 1 | FUN_008457 | contig_5 | 574565 | 575811 |
| 30 | 2 | FUN_008458 | contig_5 | 579639 | 580635 |
| 30 | 3 | FUN_008459 | contig_5 | 586984 | 590856 |
| 31 | 1 | FUN_008508 | contig_5 | 786266 | 788425 |
| 32 | 1 | FUN_008542 | contig_5 | 900917 | 903842 |
| 33 | 1 | FUN_008666 | contig_5 | 1217568 | 1220233 |
| 33 | 2 | FUN_008667 | contig_5 | 1220960 | 1221548 |
| 34 | 1 | FUN_008672 | contig_5 | 1255062 | 1258018 |
| 34 | 2 | FUN_008673 | contig_5 | 1259749 | 1260180 |
| 34 | 3 | FUN_008674 | contig_5 | 1265487 | 1266488 |
| 35 | 1 | FUN_008731 | contig_5 | 1451733 | 1455525 |
| 35 | 2 | FUN_008732 | contig_5 | 1455836 | 1457377 |
| 36 | 1 | FUN_008735 | contig_5 | 1467972 | 1471183 |
| 36 | 2 | FUN_008736 | contig_5 | 1474587 | 1475675 |
| 36 | 3 | FUN_008737 | contig_5 | 1476238 | 1479174 |
| 37 | 1 | FUN_008939 | contig_6 | 19037 | 20619 |
| 37 | 2 | FUN_008940 | contig_6 | 21218 | 22698 |
| 37 | 3 | FUN_008941 | contig_6 | 23061 | 23729 |
| 38 | 1 | FUN_009001 | contig_6 | 211855 | 212660 |
| 39 | 1 | FUN_009037 | contig_6 | 309627 | 311035 |
| 39 | 2 | FUN_009038 | contig_6 | 311145 | 319240 |
| 39 | 3 | FUN_009039 | contig_6 | 322748 | 324088 |
| 40 | 1 | FUN_009062 | contig_6 | 388785 | 392007 |
| 41 | 1 | FUN_009113 | contig_6 | 554402 | 555379 |
| 41 | 2 | FUN_009114 | contig_6 | 557503 | 558565 |
| 41 | 3 | FUN_009115 | contig_6 | 559127 | 560212 |
| 41 | 4 | FUN_009116 | contig_6 | 560550 | 561584 |
| 42 | 1 | FUN_009122 | contig_6 | 584305 | 584850 |
| 42 | 2 | FUN_009123 | contig_6 | 585737 | 586810 |
| 43 | 1 | FUN_009498 | contig_6 | 1692341 | 1693179 |
| 43 | 2 | FUN_009499 | contig_6 | 1693932 | 1694471 |
| 44 | 1 | FUN_009656 | contig_6 | 2157292 | 2162467 |
| 45 | 1 | FUN_009760 | contig_7 | 50438 | 52066 |
| 45 | 2 | FUN_009761 | contig_7 | 53651 | 54076 |
| 46 | 1 | FUN_009822 | contig_7 | 221614 | 222329 |
| 47 | 1 | FUN_009939 | contig_7 | 552404 | 553086 |
| 48 | 1 | FUN_010039 | contig_7 | 868261 | 869640 |
| 48 | 2 | FUN_010040 | contig_7 | 870168 | 870641 |
| 48 | 3 | FUN_010041 | contig_7 | 873495 | 874413 |
| 48 | 4 | FUN_010042 | contig_7 | 887267 | 889497 |
| 48 | 5 | FUN_010043 | contig_7 | 892080 | 892523 |
| 48 | 6 | FUN_010044 | contig_7 | 894454 | 894744 |
| 48 | 7 | FUN_010045 | contig_7 | 898693 | 899213 |
| 48 | 8 | FUN_010046 | contig_7 | 899775 | 901140 |
| 48 | 9 | FUN_010047 | contig_7 | 901394 | 904006 |
| 49 | 1 | FUN_010066 | contig_7 | 973959 | 975788 |
| 50 | 1 | FUN_010081 | contig_7 | 1004322 | 1006362 |
| 50 | 2 | FUN_010082 | contig_7 | 1009910 | 1011225 |
| 51 | 1 | FUN_010124 | contig_7 | 1143375 | 1144956 |
| 51 | 2 | FUN_010125 | contig_7 | 1145268 | 1145942 |
| 52 | 1 | FUN_010279 | contig_7 | 1590352 | 1591758 |
| 52 | 2 | FUN_010280 | contig_7 | 1592385 | 1593432 |
| 52 | 3 | FUN_010281 | contig_7 | 1594214 | 1594760 |
| 53 | 1 | FUN_010445 | contig_8 | 279253 | 281081 |
| 53 | 2 | FUN_010446 | contig_8 | 282661 | 283789 |
| 54 | 1 | FUN_010858 | contig_8 | 1537753 | 1537833 |
| 55 | 1 | FUN_011195 | contig_8 | 2509701 | 2510876 |
| 56 | 1 | FUN_011381 | contig_9 | 624991 | 626917 |
| 56 | 2 | FUN_011382 | contig_9 | 629528 | 632598 |
| 56 | 3 | FUN_011383 | contig_9 | 634039 | 635307 |
| 57 | 1 | FUN_011760 | contig_9 | 1793813 | 1797698 |
| 58 | 1 | FUN_011795 | contig_9 | 1881020 | 1882510 |
| 59 | 1 | FUN_011980 | contig_9 | 2386335 | 2387245 |
| 59 | 2 | FUN_011981 | contig_9 | 2387727 | 2387798 |
| 59 | 3 | FUN_011982 | contig_9 | 2389151 | 2390411 |
| 60 | 1 | FUN_000871 | contig_10 | 22513 | 25354 |
| 61 | 1 | FUN_000873 | contig_10 | 53436 | 54047 |
| 61 | 2 | FUN_000874 | contig_10 | 54084 | 54365 |
| 61 | 3 | FUN_000875 | contig_10 | 60973 | 63718 |
| 61 | 4 | FUN_000876 | contig_10 | 64073 | 64585 |
| 61 | 5 | FUN_000877 | contig_10 | 65524 | 66675 |
| 62 | 1 | FUN_000937 | contig_10 | 241868 | 243527 |
| 63 | 1 | FUN_001145 | contig_10 | 928553 | 929510 |
| 64 | 1 | FUN_001291 | contig_11 | 397789 | 399615 |
| 65 | 1 | FUN_001302 | contig_11 | 421970 | 422777 |
| 65 | 2 | FUN_001303 | contig_11 | 424287 | 429317 |
| 65 | 3 | FUN_001304 | contig_11 | 431567 | 433149 |
| 65 | 4 | FUN_001305 | contig_11 | 434758 | 436455 |
| 66 | 1 | FUN_001658 | contig_11 | 1514059 | 1515806 |
| 66 | 2 | FUN_001659 | contig_11 | 1518081 | 1518896 |
| 67 | 1 | FUN_001752 | contig_11 | 1809993 | 1813418 |
| 68 | 1 | FUN_001771 | contig_12 | 22513 | 25354 |
| 69 | 1 | FUN_001802 | contig_12 | 183812 | 184210 |
| 69 | 2 | FUN_001803 | contig_12 | 184456 | 185292 |
| 69 | 3 | FUN_001804 | contig_12 | 188102 | 189757 |
| 69 | 4 | FUN_001805 | contig_12 | 189911 | 191184 |
| 69 | 5 | FUN_001806 | contig_12 | 191983 | 192426 |
| 70 | 1 | FUN_001811 | contig_12 | 238821 | 239337 |
| 70 | 2 | FUN_001812 | contig_12 | 239439 | 240806 |
| 70 | 3 | FUN_001813 | contig_12 | 242086 | 242826 |
| 70 | 4 | FUN_001814 | contig_12 | 242916 | 244094 |
| 70 | 5 | FUN_001815 | contig_12 | 244784 | 245800 |
| 70 | 6 | FUN_001816 | contig_12 | 246209 | 246555 |
| 71 | 1 | FUN_001874 | contig_12 | 423848 | 425967 |
| 71 | 2 | FUN_001875 | contig_12 | 428264 | 428956 |
| 72 | 1 | FUN_002028 | contig_12 | 908231 | 909028 |
| 72 | 2 | FUN_002029 | contig_12 | 912956 | 914293 |
| 72 | 3 | FUN_002030 | contig_12 | 914300 | 915271 |
| 72 | 4 | FUN_002031 | contig_12 | 916728 | 917944 |
| 72 | 5 | FUN_002032 | contig_12 | 922486 | 922735 |
| 72 | 6 | FUN_002033 | contig_12 | 923912 | 926237 |
| 73 | 1 | FUN_002037 | contig_12 | 945992 | 947825 |
| 73 | 2 | FUN_002038 | contig_12 | 948763 | 950547 |
| 73 | 3 | FUN_002039 | contig_12 | 951230 | 952483 |
| 73 | 4 | FUN_002040 | contig_12 | 952980 | 953666 |
| 73 | 5 | FUN_002041 | contig_12 | 954303 | 955091 |
| 73 | 6 | FUN_002042 | contig_12 | 955125 | 956338 |
| 73 | 7 | FUN_002043 | contig_12 | 957902 | 959070 |
| 74 | 1 | FUN_002070 | contig_12 | 1021174 | 1022091 |
| 74 | 2 | FUN_002071 | contig_12 | 1022311 | 1023592 |
| 74 | 3 | FUN_002072 | contig_12 | 1025947 | 1027034 |
| 74 | 4 | FUN_002073 | contig_12 | 1030399 | 1035789 |
| 75 | 1 | FUN_002198 | contig_12 | 1367280 | 1370830 |
| 75 | 2 | FUN_002199 | contig_12 | 1371500 | 1372363 |
| 75 | 3 | FUN_002200 | contig_12 | 1372856 | 1375241 |
| 75 | 4 | FUN_002201 | contig_12 | 1375495 | 1376754 |
| 76 | 1 | FUN_002210 | contig_12 | 1402204 | 1403037 |
| 76 | 2 | FUN_002211 | contig_12 | 1406900 | 1408591 |
| 77 | 1 | FUN_002307 | contig_12 | 1685855 | 1686607 |
| 78 | 1 | FUN_002817 | contig_13 | 751236 | 752642 |
| 79 | 1 | FUN_003207 | contig_13 | 1957801 | 1959306 |
| 80 | 1 | FUN_003356 | contig_14 | 166385 | 167752 |
| 81 | 1 | FUN_003617 | contig_14 | 1008072 | 1010548 |
| 82 | 1 | FUN_003713 | contig_14 | 1294423 | 1296322 |
| 82 | 2 | FUN_003714 | contig_14 | 1296449 | 1297475 |
| 82 | 3 | FUN_003715 | contig_14 | 1298684 | 1299443 |
| 83 | 1 | FUN_003778 | contig_14 | 1485347 | 1486969 |
| 84 | 1 | FUN_004425 | contig_14 | 3505369 | 3505849 |
| 84 | 2 | FUN_004426 | contig_14 | 3506323 | 3506880 |
| 84 | 3 | FUN_004427 | contig_14 | 3507305 | 3507842 |
| 84 | 4 | FUN_004428 | contig_14 | 3507950 | 3508459 |
| 84 | 5 | FUN_004429 | contig_14 | 3509600 | 3510880 |
| 84 | 6 | FUN_004430 | contig_14 | 3511459 | 3512664 |
| 85 | 1 | FUN_004456 | contig_14 | 3580893 | 3583244 |
| 85 | 2 | FUN_004457 | contig_14 | 3584738 | 3585751 |
| 85 | 3 | FUN_004458 | contig_14 | 3586065 | 3586586 |
| 86 | 1 | FUN_004552 | contig_14 | 3858920 | 3861925 |
| 87 | 1 | FUN_004554 | contig_14 | 3867775 | 3869635 |
| 87 | 2 | FUN_004555 | contig_14 | 3873645 | 3874409 |
| 88 | 1 | FUN_004681 | contig_15 | 103124 | 104128 |
| 89 | 1 | FUN_004683 | contig_15 | 118078 | 120208 |
| 90 | 1 | FUN_004789 | contig_16 | 77093 | 78633 |
| 90 | 2 | FUN_004790 | contig_16 | 79277 | 79939 |
| 91 | 1 | FUN_004796 | contig_16 | 93016 | 94299 |
| 92 | 1 | FUN_004893 | contig_16 | 355538 | 357439 |
| 92 | 2 | FUN_004894 | contig_16 | 362381 | 364638 |
| 93 | 1 | FUN_005083 | contig_16 | 952999 | 954027 |
| 93 | 2 | FUN_005084 | contig_16 | 955429 | 955841 |
| 93 | 3 | FUN_005085 | contig_16 | 955907 | 956625 |
| 93 | 4 | FUN_005086 | contig_16 | 957151 | 958877 |
| 94 | 1 | FUN_005142 | contig_17 | 91521 | 92605 |
| 94 | 2 | FUN_005143 | contig_17 | 94262 | 95053 |
| 94 | 3 | FUN_005144 | contig_17 | 96106 | 96720 |
| 94 | 4 | FUN_005145 | contig_17 | 96955 | 97893 |
| 95 | 1 | FUN_005186 | contig_17 | 206274 | 207917 |
| 95 | 2 | FUN_005187 | contig_17 | 208366 | 209049 |
| 95 | 3 | FUN_005188 | contig_17 | 209737 | 210643 |
| 96 | 1 | FUN_005432 | contig_19 | 93549 | 94204 |
| 97 | 1 | FUN_005449 | contig_19 | 145830 | 147021 |
| 97 | 2 | FUN_005450 | contig_19 | 150044 | 151470 |
| 97 | 3 | FUN_005451 | contig_19 | 152169 | 152499 |
| 98 | 1 | FUN_006388 | contig_22 | 40204 | 41315 |
| 99 | 1 | FUN_006391 | contig_23 | 9400 | 12808 |
| 100 | 1 | FUN_006443 | contig_23 | 186465 | 187340 |
| 101 | 1 | FUN_007170 | contig_25 | 195137 | 195931 |
| 101 | 2 | FUN_007171 | contig_25 | 198760 | 199203 |
