# Supplementary material for: Comparison of Two Aspergillus oryzae Genomes From Different Clades Reveals Independent Evolution of Alpha-Amylase Duplication, Variation in Secondary Metabolism Genes, and Differences in Primary Metabolism
Source: Front Microbiol. 2021 Jul 13;12:691296. doi: 10.3389/fmicb.2021.691296 (PMC8313989; doi:10.3389/fmicb.2021.691296)
Supplement: Supplementary file 2 [file Data_Sheet_2.zip › Image 2.PDF]

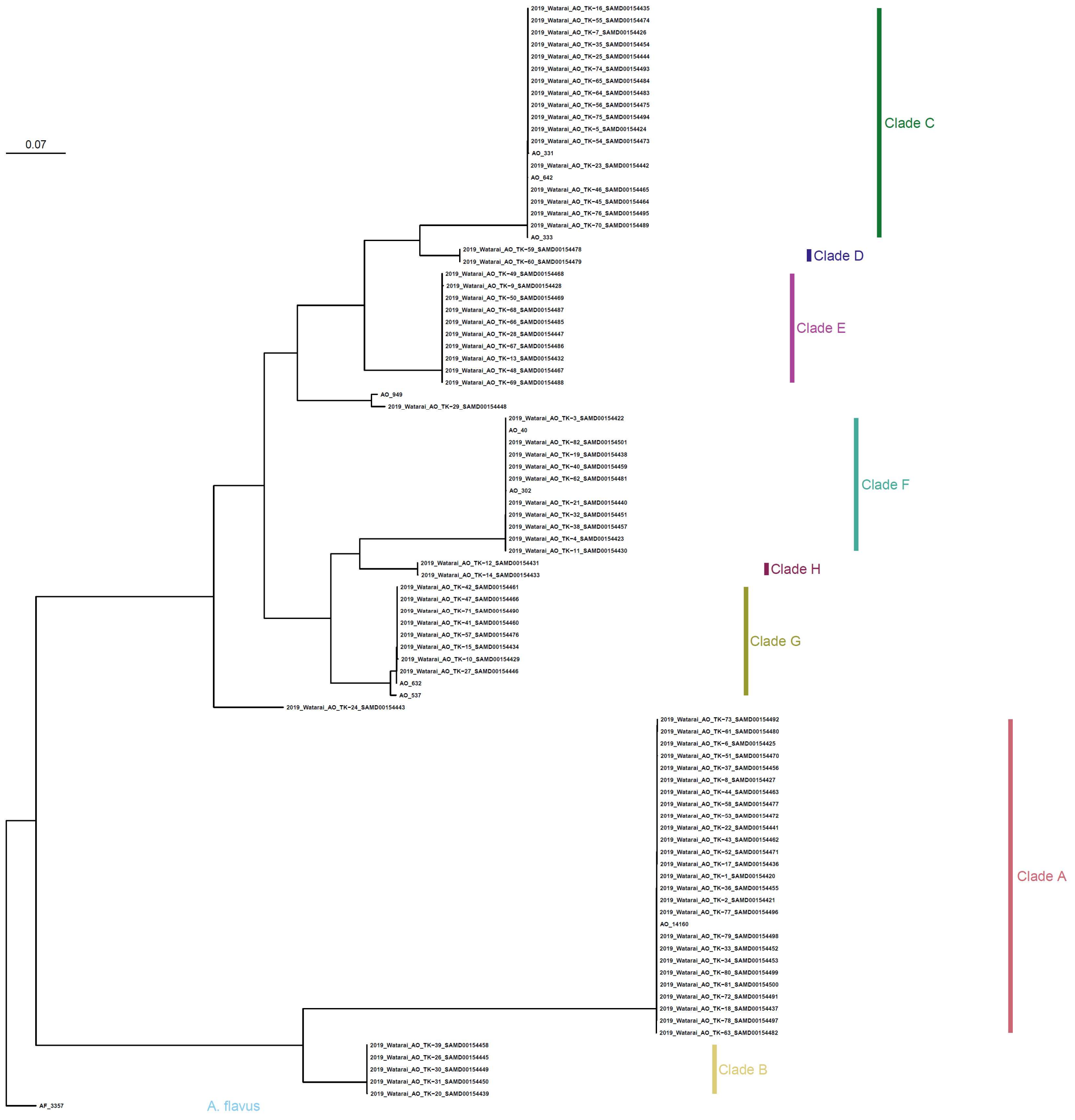

Figure S2. Phylogenetic relationship of isolates inferred from 3,340 SNP markers separated by a minimum of 10 Kb.
