# Supplementary material for: Comparison of Two Aspergillus oryzae Genomes From Different Clades Reveals Independent Evolution of Alpha-Amylase Duplication, Variation in Secondary Metabolism Genes, and Differences in Primary Metabolism
Source: Front Microbiol. 2021 Jul 13;12:691296. doi: 10.3389/fmicb.2021.691296 (PMC8313989; doi:10.3389/fmicb.2021.691296)
Supplement: Supplementary file 2 [file Data_Sheet_2.zip › Image 6.PDF]

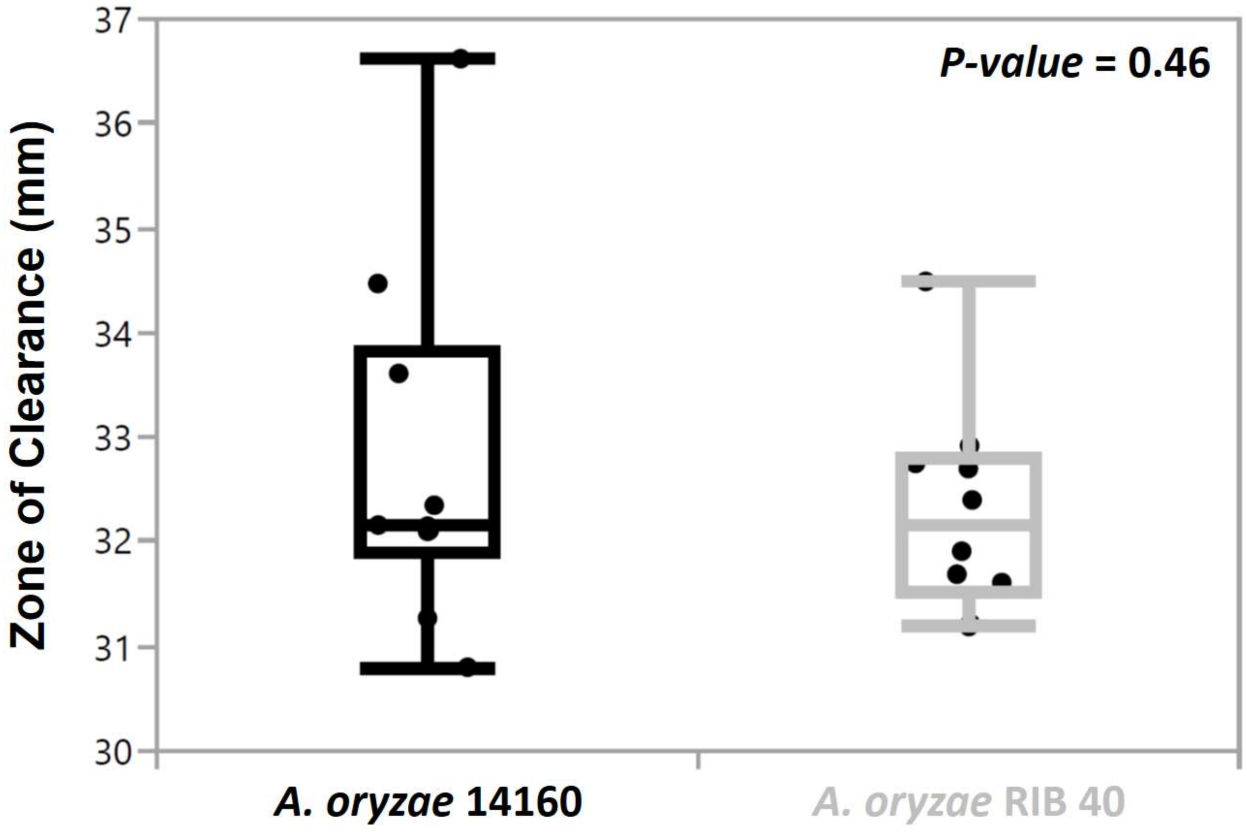

Figure S6. Proteolytic activity of *A. oryzae* 14160 and RIB 40. Proteolytic activity was measured as the zone of clearance surrounding the fungal colony (Y-axis) during growth on skim milk media, as described by Rajamani and Hilda (2018) [59]. Ten biological replicates of each strain were grown at 32°C for 72 hours. The *p-value* represents results from a student's T-test.
