# Supplementary material for: Comparison of Two Aspergillus oryzae Genomes From Different Clades Reveals Independent Evolution of Alpha-Amylase Duplication, Variation in Secondary Metabolism Genes, and Differences in Primary Metabolism
Source: Front Microbiol. 2021 Jul 13;12:691296. doi: 10.3389/fmicb.2021.691296 (PMC8313989; doi:10.3389/fmicb.2021.691296)
Supplement: Supplementary file 2 [file Data_Sheet_2.zip › Image 7.PDF]

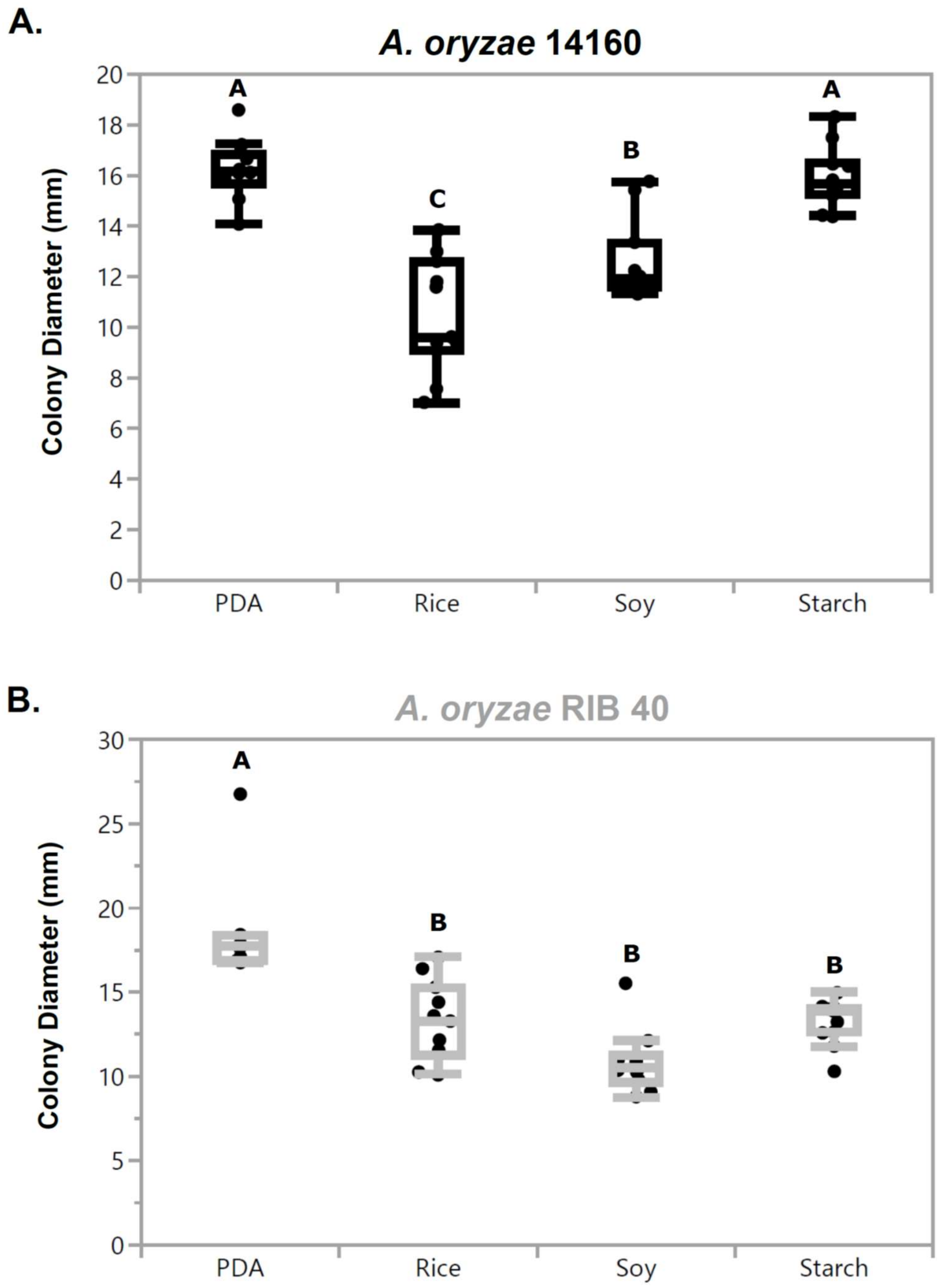

Figure S7. Growth rate (y-axis) of *A. oryzae* 14160 (A) and RIB 40 (B) on potato dextrose agar (PDA), rice agar (rice), soy agar (soy), and starch agar (starch) (x-axis). Growth rate was measured as colony diameter after 39 hours at 32° C with 10 replicates.
